# Metabolomics combined with network pharmacology reveals a role for astragaloside IV in inhibiting enterovirus 71 replication via PI3K-AKT signaling

**DOI:** 10.1186/s12967-024-05355-9

**Published:** 2024-06-10

**Authors:** JinFang Hao, Xiaoyan Zhang, Ruixian Hu, Xiufeng Lu, Hui Wang, Yuanhong Li, Kai Cheng, Qingshan Li

**Affiliations:** 1 School of Pharmaceutical, Department of Laboratory Medicine of Fenyang College, Shanxi Medical University, Taiyuan, Shanxi Province 030001 China; 2 Shanxi Key Laboratory of Innovative Drug for the Treatment of Serious Diseases Basing Chronic Inflammation, Shanxi University of Chinese Medicine, Jinzhong, 030619 China; 3 Medicinal Basic Research Innovation Center of Chronic Kidney Disease, Ministry of Education, Shanxi Medical University, Taiyuan, China

**Keywords:** Enterovirus 71, Astragaloside IV, Network pharmacology, Metabolomics, Oxidative stress response, PI3K-AKT signaling

## Abstract

**Background:**

Astragaloside IV (AST-IV), as an effective active ingredient of Astragalus membranaceus (Fisch.) Bunge. It has been found that AST-IV inhibits the replication of dengue virus, hepatitis B virus, adenovirus, and coxsackievirus B3. Enterovirus 71 (EV71) serves as the main pathogen in severe hand-foot-mouth disease (HFMD), but there are no specific drugs available. In this study, we focus on investigating whether AST-IV can inhibit EV71 replication and explore the potential underlying mechanisms.

**Methods:**

The GES-1 or RD cells were infected with EV71, treated with AST-IV, or co-treated with both EV71 and AST-IV. The EV71 structural protein VP1 levels, the viral titers in the supernatant were measured using western blot and 50% tissue culture infective dose (TCID_50_), respectively. Network pharmacology was used to predict possible pathways and targets for AST-IV to inhibit EV71 replication. Additionally, ultra-high performance liquid chromatography-high resolution mass spectrometry (UHPLC-HRMS) was used to investigate the potential targeted metabolites of AST-IV. Associations between metabolites and apparent indicators were performed via Spearman’s algorithm.

**Results:**

This study illustrated that AST-IV effectively inhibited EV71 replication. Network pharmacology suggested that AST-IV inhibits EV71 replication by targeting PI3K-AKT. Metabolomics results showed that AST-IV achieved these effects by elevating the levels of hypoxanthine, 2-ketobutyric acid, adenine, nicotinic acid mononucleotide, prostaglandin H2, 6-hydroxy-1 H-indole-3- acetamide, oxypurinol, while reducing the levels of PC (14:0/15:0). Furthermore, AST-IV also mitigated EV71-induced oxidative stress by reducing the levels of MDA, ROS, while increasing the activity of T-AOC, CAT, GSH-Px. The inhibition of EV71 replication was also observed when using the ROS inhibitor N-Acetylcysteine (NAC). Additionally, AST-IV exhibited the ability to activate the PI3K-AKT signaling pathway and suppress EV71-induced apoptosis.

**Conclusion:**

This study suggests that AST-IV may activate the cAMP and the antioxidant stress response by targeting eight key metabolites, including hypoxanthine, 2-ketobutyric acid, adenine, nicotinic acid mononucleotide, prostaglandin H2, 6-Hydroxy-1 H-indole-3-acetamide, oxypurinol and PC (14:0/15:0). This activation can further stimulate the PI3K-AKT signaling to inhibit EV71-induced apoptosis and EV71 replication.

**Graphical Abstract:**

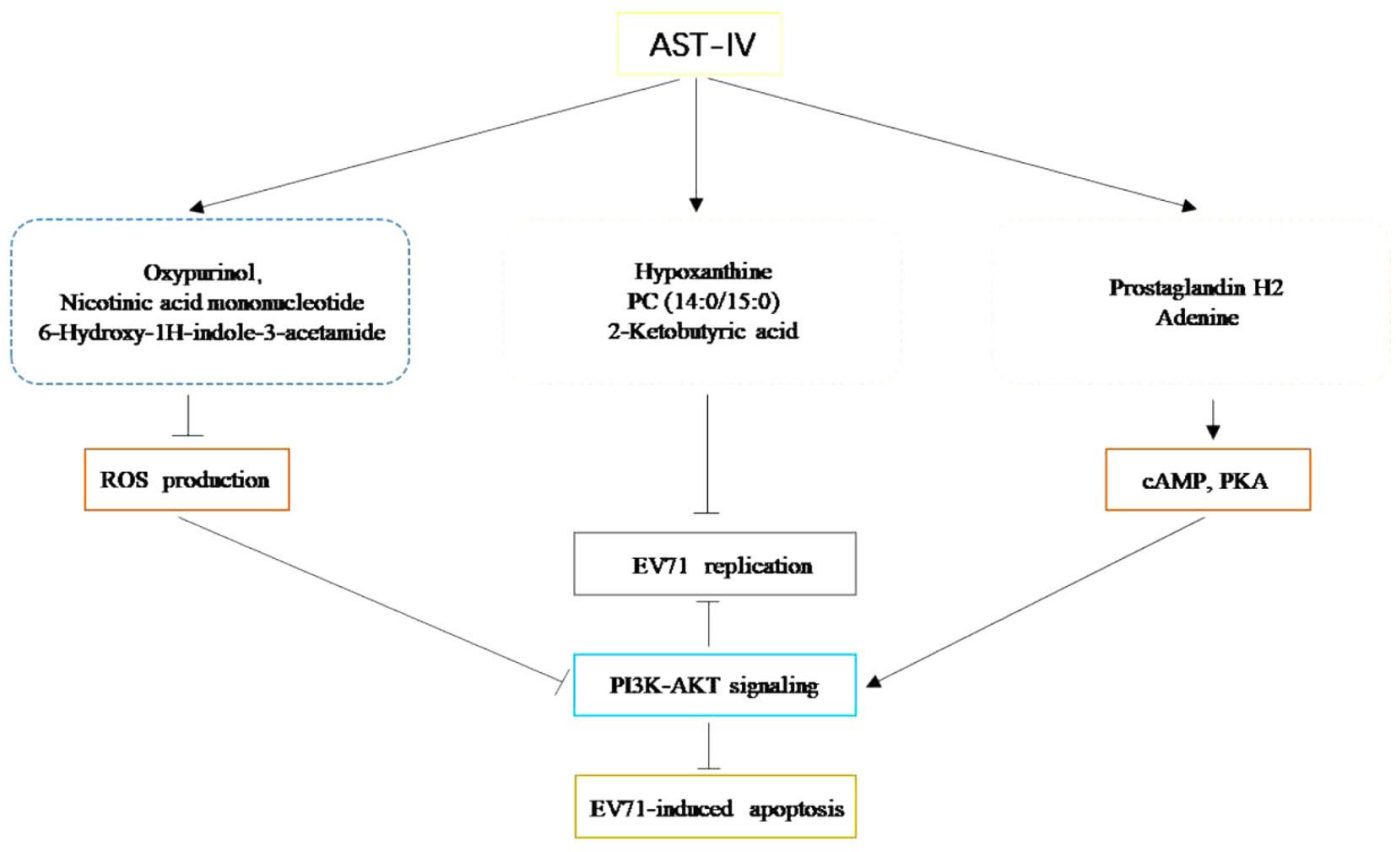

**Supplementary Information:**

The online version contains supplementary material available at 10.1186/s12967-024-05355-9.

## Introduction

Hand-foot-mouth disease (HFMD) is caused by a multitude of enteroviral infections and poses a significant public health threat to children [1]. Enterovirus 71 (EV71), a common pathogen of HFMD, is classified as an enterovirus in the family of small RNA viruses [2]. EV71 commonly infects children under the age of 5 years, and the resulting HFMD is typically self-limiting. However, a small proportion of severely ill patients can develop pulmonary edema, myocarditis, and potentially fatal encephalitis in newborns [3]. However, many clinical investigations have shown that HFMD can be mitigated by diet or dietary components.

Viral infections often disrupt the body’s oxidative stress response, leading to compromised immune function, apoptosis, inflammation, and organ and tissue dysfunction that contribute to patient pathogenesis. HBV and HIV infection induces oxidative stress damage by producing reactive oxygen species (ROS) in infected cells [4, 5], while the excessive production of ROS can induce apoptosis (Giorgio et al., 2005). Nuclear Factor E2-Related Factor 2 (Nrf2) serves a crucial role in the antioxidant stress response as a regulatory protein downstream of ROS [6]. Nrf2 activators [7] or the activation of Nrf2 [8, 9] can exhibit antiviral effects. The PI3K/AKT signaling pathway is implicated in the regulation of apoptosis, cell proliferation, and viral infection [10–12]. The use of PI3K/AKT inhibitors has been shown to promote infection by infectious spleen and kidney necrosis viruses [13], indicating that PI3K/AKT could be a potential therapeutic target for viral diseases.

Astragalus membranaceus (Fisch.) Bunge, is commonly used in traditional formulas to prevent the invasion of external pathogens by strengthening the lungs and spleen [14], treat human immunodeficiency virus (HIV) infection [15], suppress SARS-CoV-2 entry [16], protect acute kidney injury [17]. Astragaloside IV (AST-IV, chemical formula: C41H68O14, molecular weight: 785), one of the active ingredients of Astragalus membranaceus (Fisch.) Bunge, has the pharmacological effects of anti-inflammatory, immunomodulatory, antibacterial and antiviral properties [18, 19]. Recent studies have showed that AST-IV inhibits the replication of dengue virus [20], hepatitis B virus [21], adenovirus [22]. It has also been found to reduce coxsackievirus B3-induced apoptosis in cardiomyocytes [23]. However, the inhibitory effect of AST-IV on EV71 virus replication and its underlying mechanism are not yet fully understood.

Network pharmacology is a method for effectively predicting relevant targets and pathways for drug treatment of diseases to further elucidate the underlying mechanisms [24]. Metabolomics has gained significant traction in disease diagnosis, drug mechanism of action studies, and the discovery of novel biomarkers [25]. Therefore, we employed an in vitro model of EV71 infection and AST-IV treatment to investigate the anti-EV71 mechanism of AST-IV. Here, we found that AST-IV inhibited EV71 replication through the ROS-PI3K-AKT pathway.

## Materials and methods

### Cell lines and culture

Normal human gastric epithelial (GES-1) and human rhabdomyosarcoma (RD) cells were described previously (Zhang et al., 2022a). For the experiments, GES-1 cells (1 × 10^6^/well) or RD cells (1 × 10^6^/well) were seeded in 6-well plates and cultured for 12 h. Subsequently, the GES-1 cells or RD cells were infected with EV71 for 24 h–12 h or pretreated with varying concentrations of AST-IV (2.5 µg/mL, 5 µg/mL, 10 µg/mL, HPLC ≥ 98%, SA8640, Solarbio, Beijing, China), ribavirin (RBV, 2.5 µg/mL, positive control, HPLC ≥ 98%, R8370, Solarbio, Beijing, China), or N-acetylcysteine (NAC, 20 µM, HY-B0215, MedChemExpress, America) for 2 h and then infected with or without EV71 for 24 h–12 h.

## Virus and virus infection

EV71 and the 50% tissue culture infective dose (TCID_50_) [26] have been described previously [27].

### Cell viability assay

GES-1 cells or RD cells were cultured in 96-well plates (1 × 10^4^/well). Subsequently, the GES-1 cells or RD cells were infected with EV71, treated with AST-IV, or treated with EV71 and AST-IV. GES-1 or RD cell viability was determined using a Cell Counting Kit-8 (CCK-8) kit (C0038, Beyotime, Shanghai, China) according to the manufacturer’s protocol.

### PI staining

4 µM propyl iodide (PI, ST511, Beyotime, Shanghai, China) was added to 125 µL of fresh DMEM and cocultured with the GES-1 cells in an incubator for 30 min. Then, 1 µL of Hoechst 33,342 (C1027, Beyotime, Shanghai, China) was added to 100 µL of fresh DMEM and cocultured with the GES-1 cells in an incubator for 10 min. The cells were then photographed using an inverted fluorescence microscope (Nikon, Japan).

### LC‒MS/MS method for GES-1 cell metabolomics

After slowly thawing the treated cells (approximately 10^^7^) at 4 °C, 1000 µL of the extraction solution (MeOH: ACN: H2O, 2:2:1 (v/v)) was added to a centrifuge tube and mixed by vortexing for 30 s. Subsequently, the GES-1 cells were frozen in liquid nitrogen, thawed at room temperature, and vortexed for 30 s and this process was repeated three times. The cells were then sonicated for 10 min on ice and kept at -40 ℃ for 1 h. The cell lysate supernatant was collected in a fresh glass vial for analysis. The supernatant was stored in a 4 °C autosampler, and 2 µL of each sample was analysed. MS/MS spectra were acquired using an Orbitrap Exploris 120 mass spectrometer controlled by Xcalibur software (Xcalibur, Thermo).

### Measurements of intracellular ROS

GES-1 cells or RD cells were incubated in DMEM containing 10 µM DCFH-DA without FBS (S0033S, Beyotime, Shanghai, China) for 30 min in an incubator. After washing with PBS, the GES-1 cells or RD cells were photographed using an inverted fluorescence microscope (Nikon, Japan). The green fluorescence intensity was measured using ImageJ software.

### Determination of the antioxidant activity of GES-1 cells and RD cells

GES-1 cells or RD cells were collected and disrupted by sonication. The levels of malondialdehyde (MDA; A003-4-1, Jiancheng, Nanjing, China), and the activity of total antioxidant capacity (T-AOC; A015-2-1, Jiancheng, Nanjing, China), catalase (CAT; S0051, Beyotime, Shanghai, China), glutathione peroxidase (GSH-Px; S0056, Beyotime, Shanghai, China), and superoxide dismutase (SOD; A001-3-1, Jiancheng, Nanjing, China) were detected using commercially available kits.

### Network pharmacology analysis

Potential targets of AST-IV were collected from the SwissTargetPrediction (http://www.swisstargetprediction.ch/) and TargetNet (http://targetnet.scbdd.com/) databases. Protein names were converted to gene names in the UniProts database. We entered the keyword " hand foot mouth disease " in the GeneCards (https://www.genecards.org/), DisGenet (https://www.disgenet.org/), and OMIM (https://www.omim.org/) databases to identify HFMD-related therapeutic targets. We also performed a collection of RNA-sequencing (GSE15323) from the Gene Expression Omnibus (GEO) database following EV71 infection and the DEGs were screened by Gene Expression Omnibus (GEO) 2R analysis with an adjustable *P* < 0.05 and a |log2-fold change (FC)| > 1.0. The HFMD targets and AST-IV targets were entered into the online platform (https://www.bioinformatics.com.cn/), and the common targets were identified using a Venn diagram. Protein-protein interaction (PPI) networks of common genes were constructed using the STRING database (https://cn.string-db.org/) with interaction scores > 0.4. Cytoscape 3.9.0 was used to visualize the PPI networks. Fill colors, heights, and widths were mapped continuously by degree. PPI network construction of key genes was performed and visualized using Metascape (https://metascape.org/). Gene Ontology (GO) functional enrichment and Kyoto Encyclopedia of Genes and Genomes (KEGG) pathway enrichment were analyzed and visualized using an online platform (https://www.bioinformatics.com.cn/, https://metascape.org/). Hub genes were selected by the Cytoscape 3.9.0 plugin cytoHubba [28], and the most critical hub genes were identified by the following algorithms: closeness, MCC, degree, MNC, radiality, stress, and EPC.

### Western blotting assays

GES-1 cell proteins were extracted using Western and IP lysis buffer (P0013, Beyotime, Shanghai, China) containing phosphatase (P1086, Beyotime, Shanghai, China) and protease (P1005, Beyotime, Shanghai, China) inhibitors. The supernatant from the cell lysate was collected by centrifugation at 13,800 × g for 15 min at 4 °C, and the protein concentrations were quantified using a BCA kit (Boster, Wuhan, China). Total proteins were detected by Western blot and the nitrocellulose (NC) membrane was incubated with phosphorylated PI3K (p-PI3K, 1:1000, CY6428, Abways), VP1 (1:1000, PAB7631-D01P, Abnova), t-AKT (t-AKT, 1:1000, AA326, Beyotime), cleaved PARP (1:1000, AF1567, Beyotime), total PI3K (t-PI3K, 1:400, 4257T, Cell Signaling), cleaved caspase-3 (1:1000, WL01992, Wanlei), phosphorylated AKT (p-AKT, 1:1000, CY6569, Abways), and GAPDH (1:5000, A00227-1, Boster).

### Molecular docking

The crystal structures of the AKT1 (PDB ID: 1h10) and PIK3R1 (PDB ID: 1pbw) proteins were downloaded from the Protein Data Bank (PDB, https://www.rcsb) database, and the structure of AST-IV was downloaded from the PubChem database (https://pubchem.ncbi.nlm.nih.gov/). Docking validation was performed using AutoDock 1.5.6 software. During the molecular docking process involving AKT1, PIK3R1 and AST-IV, a grid was created with dimensions x = 56.3333333333, y = 52.0, z = 65.0 or x = 98.0, y = 100.0, z = 116.0 respectively, and a number of steps set to 10. The grid was defined to encompass the proteins for blind docking. Autodock generated 9 complexes as a result of the docking simulation. All of the above docking is semi-flexible. The docking results were visualized using PyMOL software.

### Statistical analysis

All the data are presented as the mean ± standard deviation (SD). Statistical analysis was performed using GraphPad Prism 8.0.2 software (CA, USA). Comparisons between two groups or multiple groups were assessed using unpaired Student’s t‑test or one‑way ANOVA with Tukey’s post hoc test, respectively. Data with unequal variances were analyzed by Welch’s test with the Games–Howell post hoc test. *p* < 0.05 was considered to indicate statistical significance. Differential metabolites were identified using a combination of the variable importance in projection (VIP) value from the OPLS-DA model and P values from Student’s t test on normalized peak areas. VIP ≥ 1 and *p* < 0.05were considered significantly altered metabolites.

## Results

### AST-IV inhibits EV71 replication

**Fig. 1:**
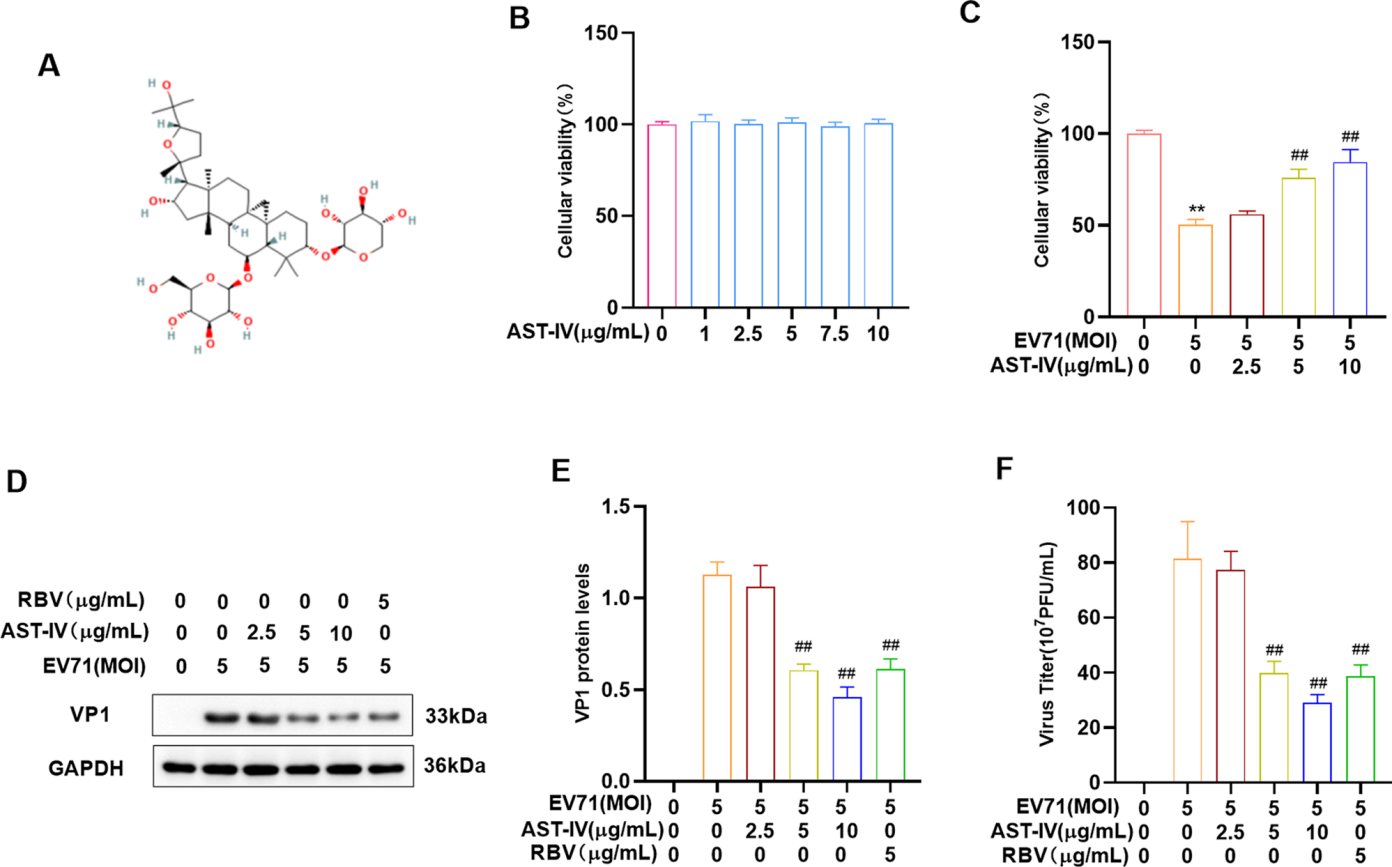
AST-IV inhibits EV71 replication. (**A**) Chemical structure of AST-IV. (**B, C**) A CCK-8 assay was performed to measure the viability of GES-1 cells in different treatment groups. (**D, E**) Western blotting was used to analyze the levels of the viral structural protein VP1 in the different treatment groups. (**F**) The viral titers in the supernatants of different treatment groups were determined by TCID50 assay. The data are presented as the mean ± SD (*n* = 3) of at least three independent experiments. ****P < 0.0001 compared with the control group; #*P* < 0.05, ##*P* < 0.01, and ###*P* < 0.001 compared with the EV71-infected group

To investigate the effect of AST-IV on EV71 replication, GES-1 cells were treated with AST-IV. AST-IV at 0–10 µg/mL had no significant effect on cell viability (Fig. 1B). Next, GES-1 cells or RD cells were pretreated with AST-IV for 2 h and then infected with EV71 for 24 h–12 h. Cell viability was determined by the CCK8 assay, viral structural protein VP1 levels were determined by Western blotting, and viral titers in the supernatants were assayed through the TCID_50_ method. The results showed that AST-IV increased the viability of EV71-infected GES-1 cells or RD cells (Fig. 1C, Fig S1). In addition, the levels of VP1 protein (Fig. 1D, E) and viral titers in the supernatant (Fig. 1F) were reduced in AST-IV- or RBV-treated and EV71-infected GES-1 cells, indicating a decrease in viral replication. Based on these results, it can be concluded that AST-IV effectively inhibited the EV71 replication.

### Effects of AST-IV on EV71-induced apoptosis

**Fig. 2:**
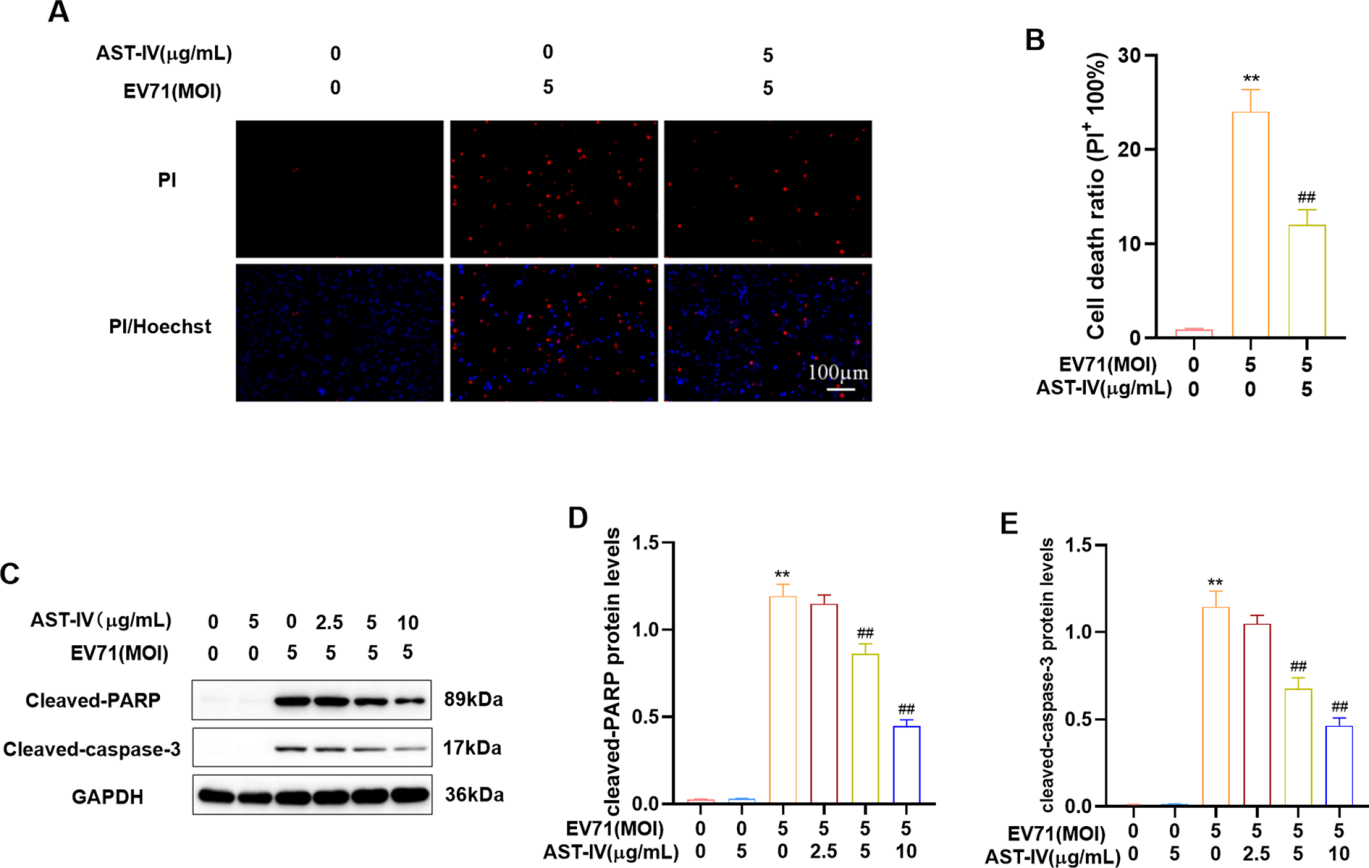
Effects of AST-IV on EV71-induced apoptosis. (**A, B**) Fluorescence images of EV71-infected GES-1 cells were obtained in the presence of AST-IV for 2 h-prior. The cells were stained with PI. (**C-E**) Western blot analysis of the apoptosis marker proteins cleaved PARP and cleaved caspase-3 in EV71-infected GES-1 cells in the presence of AST-IV. The data are presented as the mean ± SD (*n* = 3) of at least three independent experiments. ****P < 0.0001 compared to thecontrol group; ##*P* < 0.01, ###*P* < 0.001, ####*P* < 0.01 compared to the EV71-infected group

Oxidative stress can activate apoptosis, and inhibition of oxidative stress protects cells from cytotoxicity and apoptosis [29, 30]. To investigate the protective effects of AST-IV on EV71-induced apoptosis, we performed PI staining to assess cell viability and Western blot to measure cleaved PARP and cleaved caspase-3 levels. The results indicated that EV71 infection significantly increased the number of PI-positive cells. However, AST-IV treatment effectively reduced it, suggesting that AST-IV has a protective effect against EV71-induced cytotoxicity (Fig. 2A, B). Furthermore, we found that cleaved PARP and cleaved caspase-3 levels were significantly increased in EV71-infected GES-1 cells, indicating the activation of apoptosis. However, AST-IV inhibited the degradation of the PARP and caspase-3 proteins induced by EV71 infection, suggesting that AST-IV can protect against EV71-induced apoptosis (Fig. 2C-E). Our results demonstrated that AST-IV protects against EV71-induced apoptosis in GES-1 cells.

### Analysis of the metabolic profile of EV71-infected and AST-IV-treated GES-1 cells

**Fig. 3:**
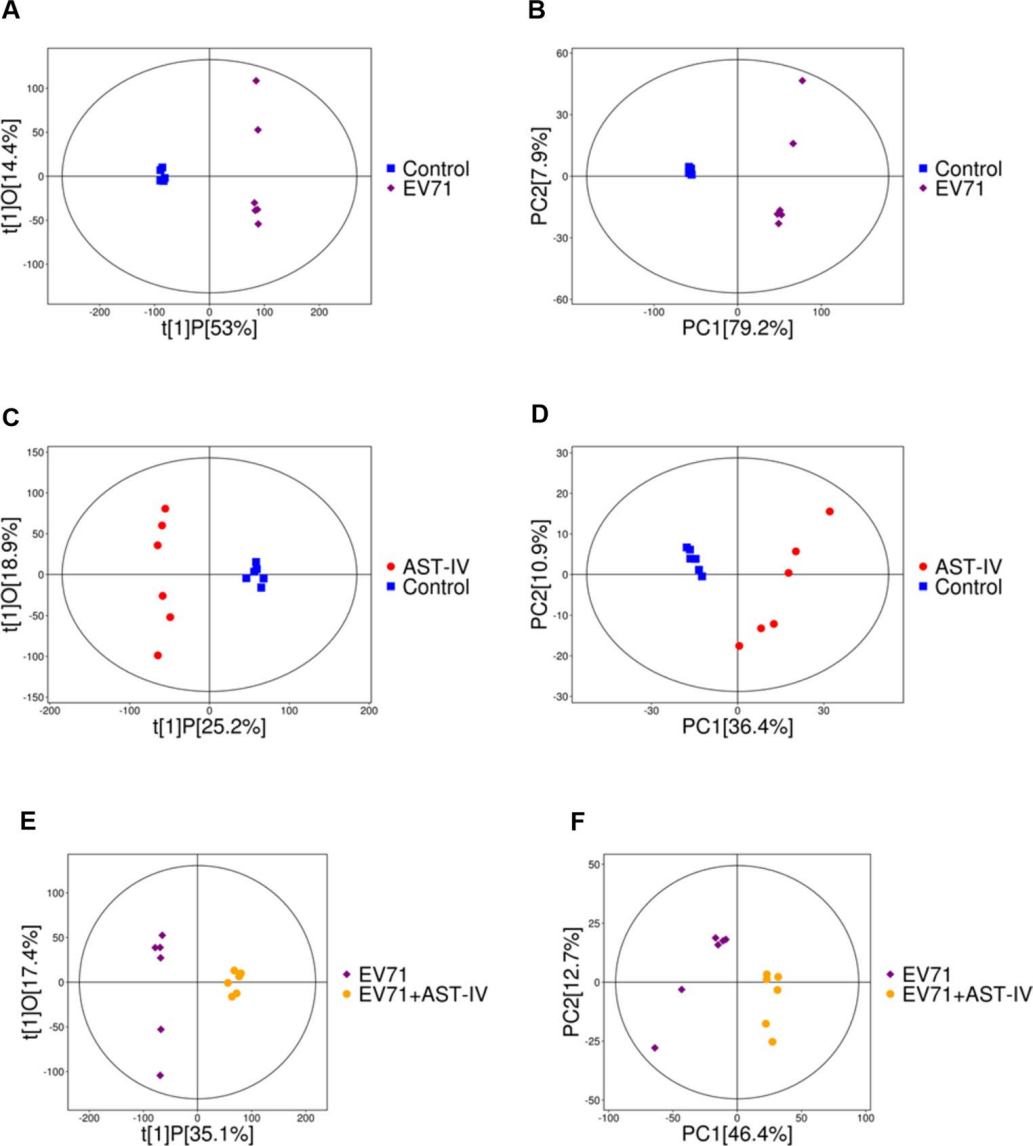
OPLS-DA and PCA analysis of LC-MS/MS of GES-1 cells. (**A**) OPLS-DA and (**B**) PCA score plots of the control group and EV71 group. (**C**) OPLS-DA and (**D**) PCA score plots of the control group and AST-IV group. (**E**) OPLS-DA and (**F**) PCA score plots of the EV71 group and EV71 + AST-IV group

**Fig. 4:**
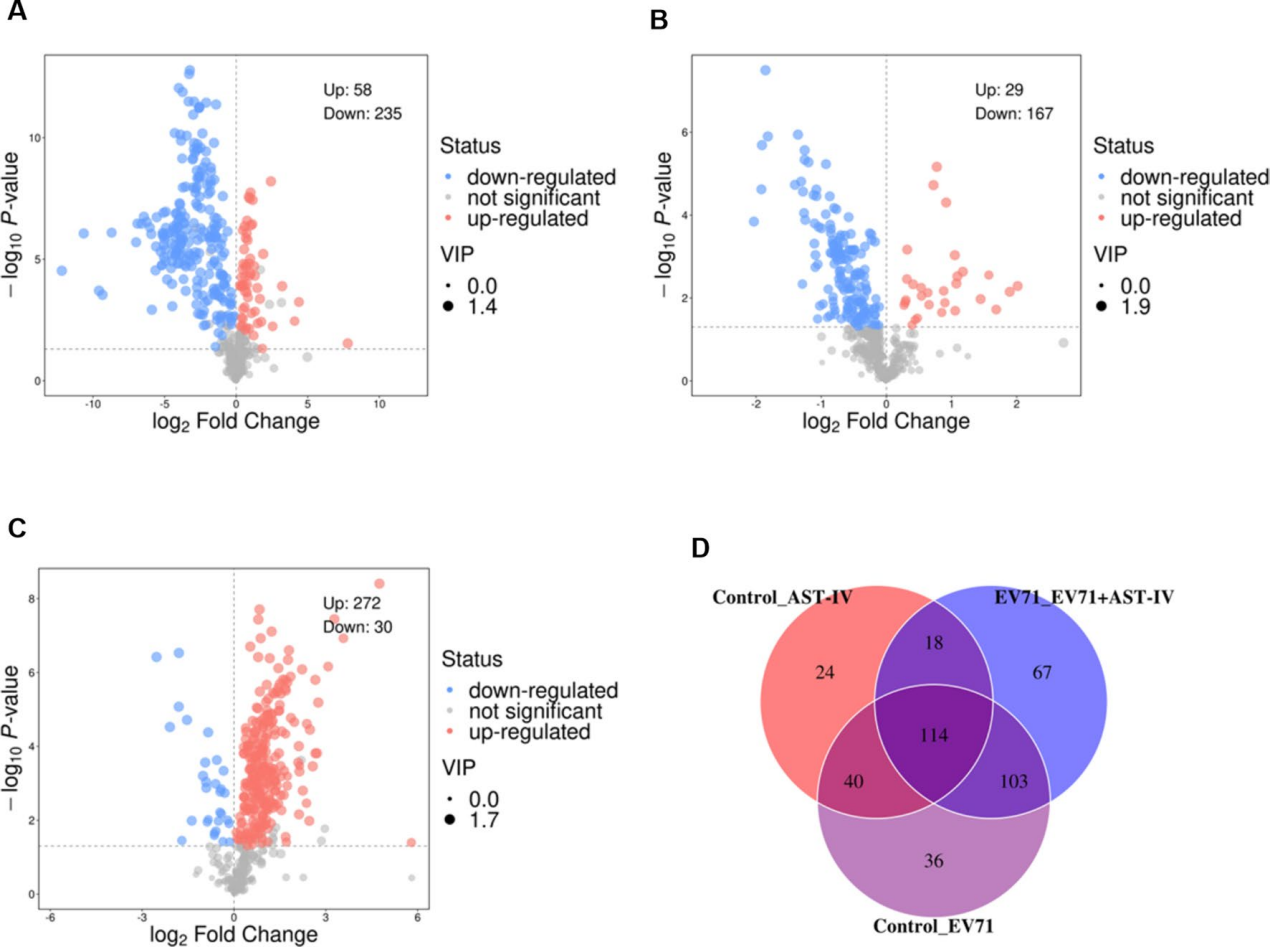
Analysis of differential metabolites in the four groups. (**A**) The volcano plot shows the differential metabolite levels between the control group and the EV71 group. (**B**) The volcano plot shows the differential metabolite levels between the control group and the AST-IV group. (**C**) The volcano plot shows the differential metabolite levels between the EV71 group and the EV71 + AST-IV group. (**D**) Venn diagram showing the overlapping metabolites from the control group, EV71 group, AST-IV group and EV71 + AST-IV group

**Fig. 5:**
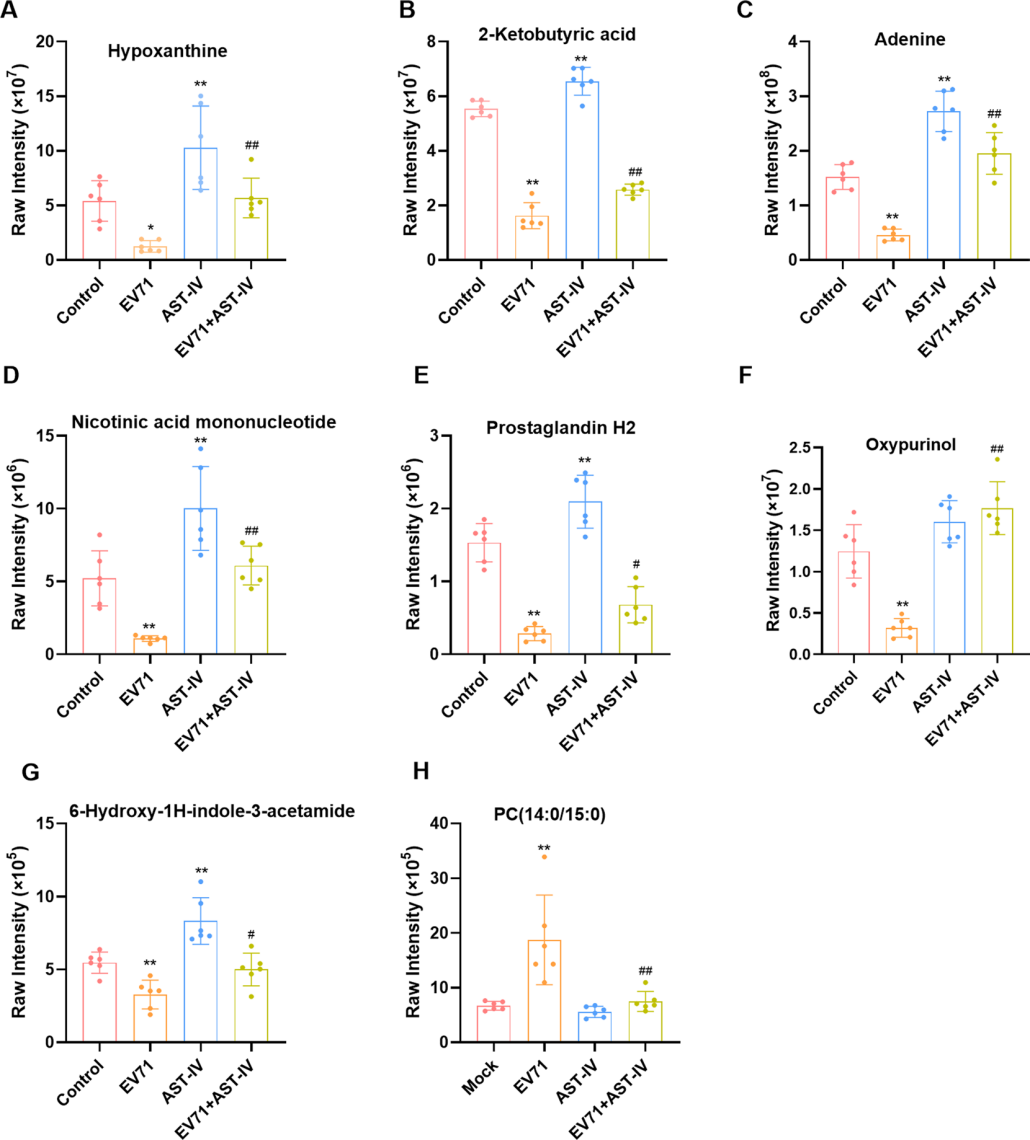
The relative peak regions of possible GES-1 cell indicators associated with EV71 infection that could be regulated by AST-IV. The data are shown as the mean ± SEM for GES-1 cells from each group (*n* = 6). *P < 0.05, **P < 0.01, ***P < 0.001, ****P < 0.0001 compared to the control group; #*P* < 0.05, ##*P* < 0.01, ###*P* < 0.001, ####*P* < 0.0001 compared to the EV71-infected group

To better understand how AST-IV exerts its anti-EV71 effects by modulating metabolites, we investigated the metabolomics of GES-1 cells. We observed an apparent separation in the OPLS-DA (Fig. 3A, C, E) and PCA (Fig. 3B, D, F) score plots among the Control, EV71, AST-IV and EV71 + AST-IV groups. The metabolic profiles of GES-1 cells were significantly different among the four groups. We used volcano and Venn diagrams to show the intrinsic connections of different metabolites. There were 293 metabolites with significant differences between the control group and the EV71 group (Fig. 4A), 196 metabolites between the control group and the AST-IV group (Fig. 4B), and 302 metabolites between the EV71 group and the AST-IV and EV71 group (Fig. 4C). Notably, we identified a total of 114 common differential metabolites among the four groups (Fig. 4D). Among these metabolites, eight were significantly altered in GES-1 cells treated with AST-IV alone. These metabolites included hypoxanthine, 2-ketobutyric acid, adenine, nicotinic acid mononucleotide, prostaglandin H2, oxypurinol, 6-hydroxy-1 H-indole-3-acetamid, and PC (14:0/15:0) (Fig. 5A-H). Furthermore, the metabolite PC (14:0/15:0) decreased (Fig. 5H), whereas hypoxanthine, 2-ketobutyric acid, adenine, nicotinic acid mononucleotide, prostaglandin H2, oxypurinol, and 6-hydroxy-1 H-indole-3-acetamid (Fig. 5A-G) increased in the AST-IV group compared to the control group. Furthermore, AST-IV treatment of EV71-infected cells restored the levels of these metabolites to essentially the same levels as those in the control group. Specifically, AST-IV increased the levels of hypoxanthine, adenine, nicotinic acid mononucleotides, and oxypurinol, indicating its effect on nucleotide metabolism. AST-IV also increased the levels of 2-ketobutyric acid, indicating its effect on cellular energy production and the citric acid cycle. In addition, AST-IV upregulated 6-hydroxy-1 H-indol-3-acetamide, suggesting its influence on cellular growth hormone metabolism. AST-IV was found to upregulate prostaglandin H2, indicating its involvement in fatty acid metabolism. Furthermore, AST-IV downregulated PC (14:0/15:0), suggesting its impact on phospholipid metabolism. Taken together, these findings provide insight into the specific metabolites affected by AST-IV in GES-1 cells. These identified metabolites may serve as potential targets by which AST-IV inhibits EV71 infection.

### Effects of AST-IV on antioxidative activity

**Fig. 6:**
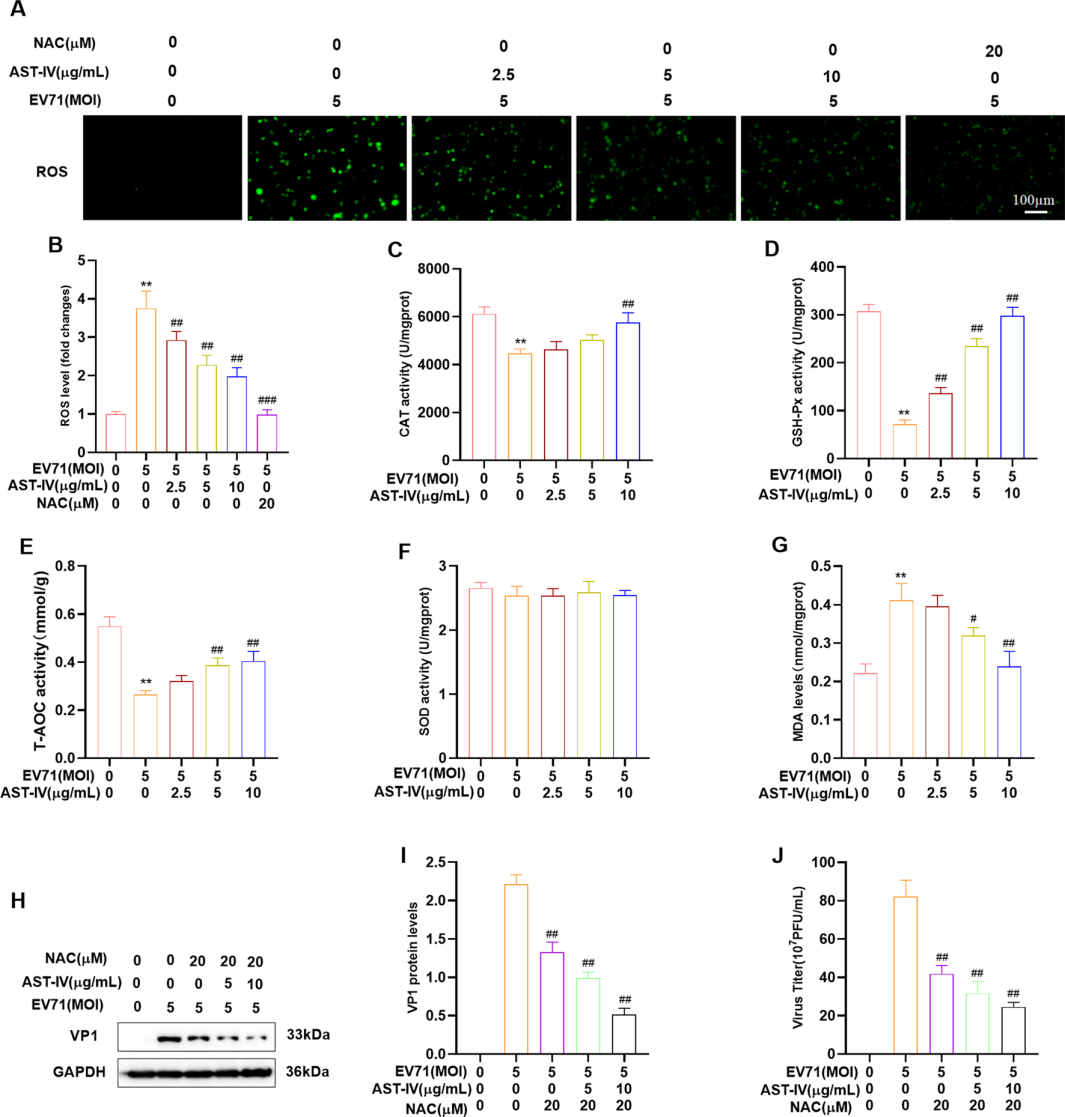
Effects of AST-IV on antioxidative activity. (**A, B**) ROS levels, (**C**) CAT activity, (**D**) GSH-Px activity, (**E**) T-AOC activity, (**F**) SOD activity, and (**G**) MDA levels were measured in different treatment groups in GES-1 cells. (**H, I**) Western blotting was used to analyze VP1 levels in EV71-infected and NAC-treated GES-1 cells. (**J**) The TCID50 assay was performed to determine the viral titers in the supernatant of EV71-infected and NAC-treated GES-1 cells. The data are presented as the mean ± SD (*n* = 3) of at least three independent experiments. **P < 0.01, ***P < 0.001, ****P < 0.0001 compared with the control group; #*P* < 0.05, ##*P* < 0.01, ###*P* < 0.001, ####*P* < 0.01 compared with the EV71-infected group

Studies have shown a strong relationship between oxidative stress and viral replication [31, 32]. Therefore, we assayed the ROS levels and oxidative stress biomarkers in different treatment groups and the results showed that AST-IV treatment reduced ROS levels and increased the activity of CAT, GSH-Px, and T-AOC while decreasing MDA levels in EV71-infected GES-1 cells or RD cells (Fig. 6A-G, Fig S2A-F). These findings indicate that AST-IV can effectively protect GES-1 cells and RD cells from EV71-induced oxidative damage. As an ROS inhibitor, we investigated the anti-EV71 effects of NAC. Our results showed that NAC treatment reduced VP1 protein levels and viral titers in the supernatants of EV71-infected GES-1 cells (Fig. 6H-J), similar to the effects of AST-IV. This finding suggested that both AST-IV and NAC may inhibit EV71 replication by reducing oxidative stress. We concluded that AST-IV exerts anti-EV71 effects by inhibiting EV71-induced oxidative stress.

### Targets of AST-IV and HFMD

**Fig. 7:**
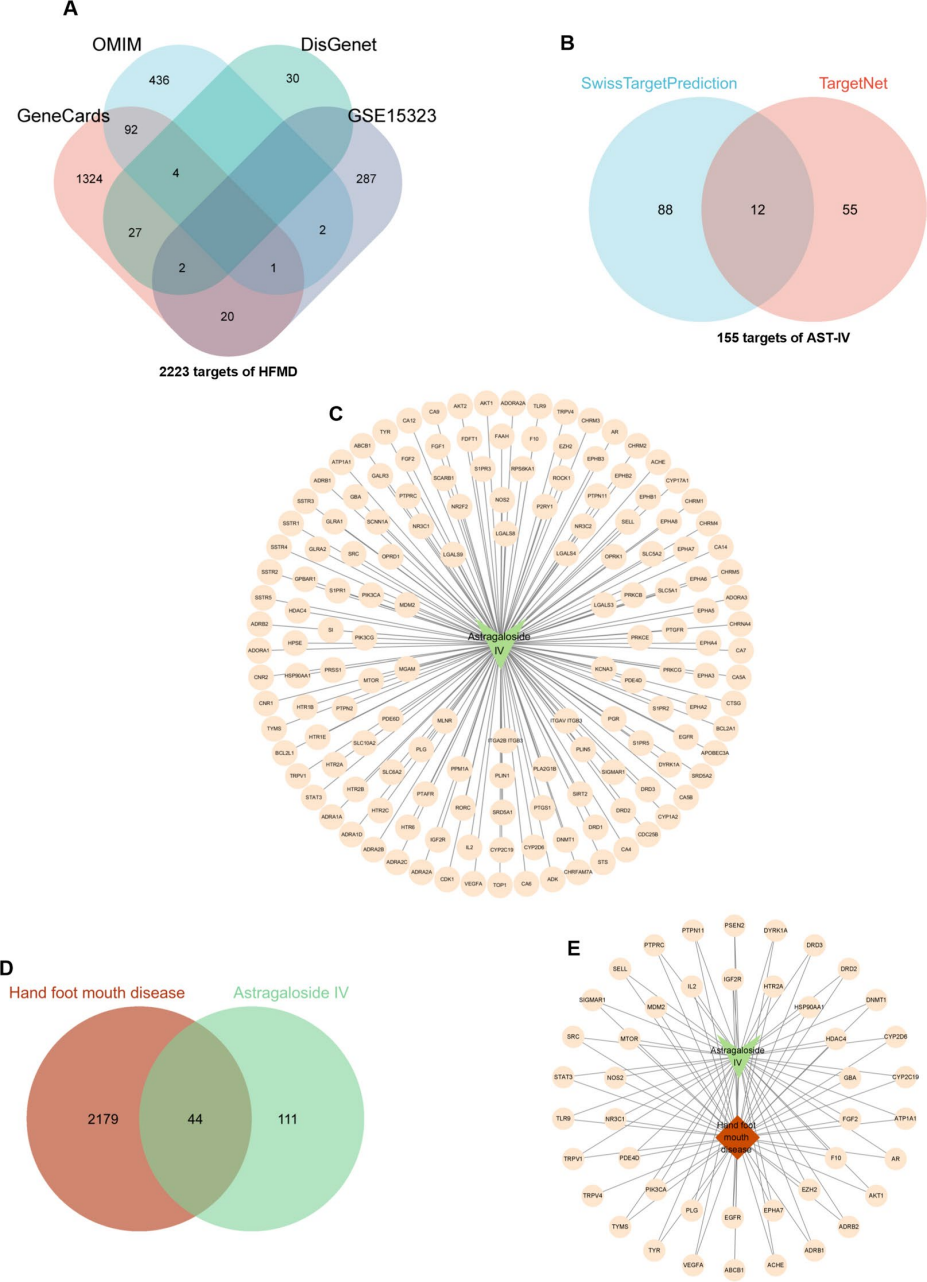
Targets of AST-IV and HFMD. (**A**) Venn diagram of HFMD-related targets. (**B**) Venn diagram of AST-IV-related targets. (**C**) Venn diagram of the common targets between AST-IV and HFMD. (**D**) Visualization of the AST-IV-target network. (**E**) Visualization of the AST-IV-common targets-HFMD network

To further investigate the mechanism of AST-IV in the treatment of HFMD at the protein level, we conducted network pharmacology analysis. Targets for HFMD were collected from 3 databases: 535 targets from the OMIM database, 63 targets from the DisGenet database, and 1470 targets from the GeneCards database. In addition, we included an RNA sequence (GSE157282) from the GEO database and used the criteria of |log_2_FC| > 1 and adjustable *P* < 0.05 to identify DEGs. By integrating the targets in the database and the DEGs from GSE157282, a total of 2223 therapeutic targets related to HFMD were identified (Fig. 7A). The AST-IV targets were collected from two databases, 100 from the Swiss TargetPrediction database and 67 from the TargetNet database, and after removing duplicate targets, a total of 100 AST-IV targets were collected (Fig. 7B). The Venn diagram showed 44 common targets between the AST-IV and HFMD (Fig. 7D). The targets were visualized as an AST-IV-target network (Fig. 7C) and an AST-IV-common targets-HFMD network (Fig. 7E) using Cytoscape 3.9.0 software. AST-IV is shown in green, HFMD in orange and the targets in yellow.

### Network construction and analysis

**Fig. 8:**
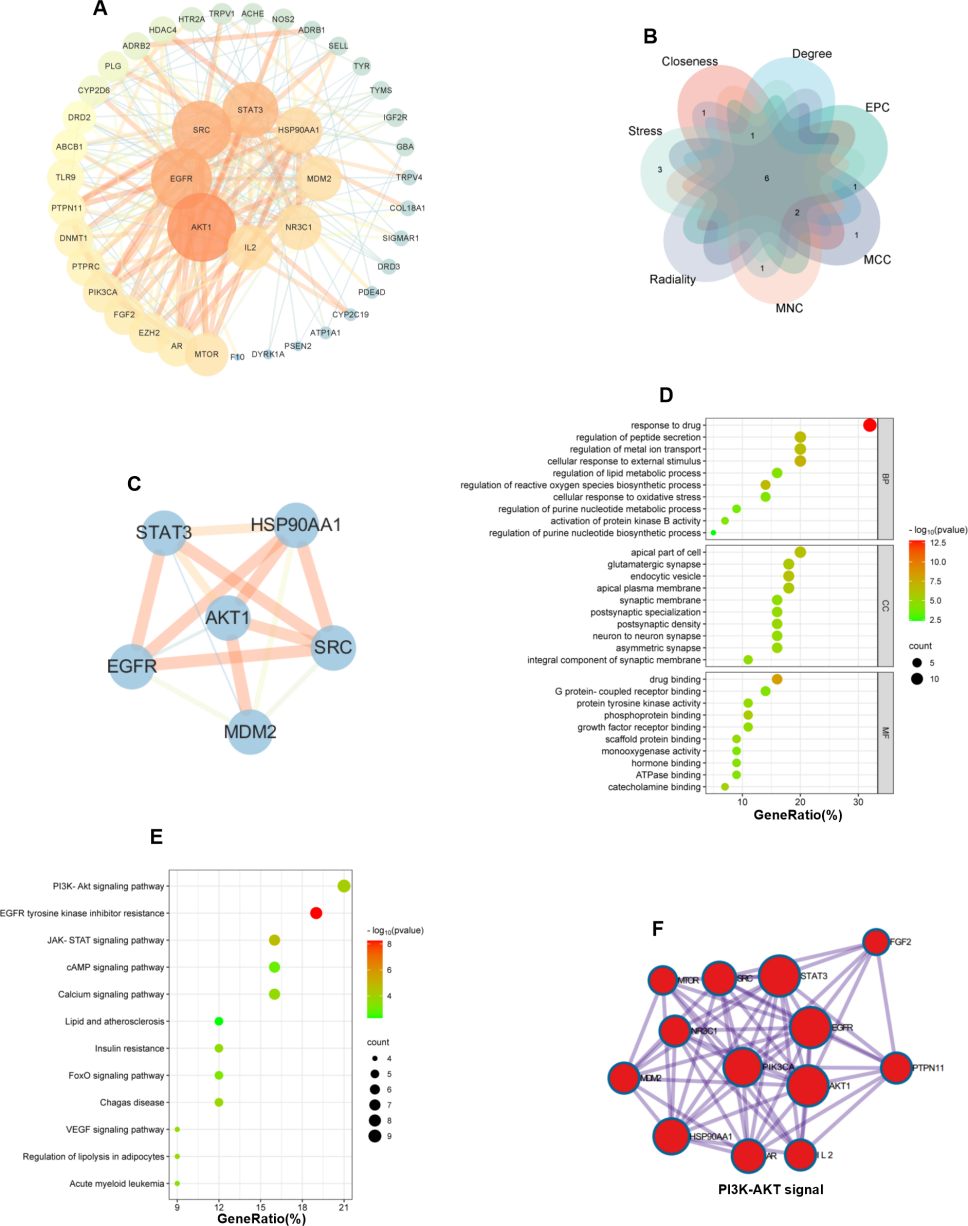
Network construction and analysis. (**A**) The PPI network was constructed using Cytoscape 3.9.0. (**B**) Venn diagram showing the 6 overlapping hub genes screened by seven algorithms. (**C**) The PPI network of the 6 overlapping hub genes. (**D**) GO enrichment analysis. (**E**) KEGG enrichment analysis. (**F**) The most significant pathways were screened by Metascape

We also constructed a PPI network of 44 common targets by using Cytoscape 3.9.0 to explore the interactions between these potential targets and screened the top 10 ranked hub genes by degree value, which were AKT1, EGFR, SRC, STAT3, HSP90AA1, IL2, MDM2, NR3C1, MTOR, and AR (Fig. 8A). We also screened the top 10 ranked hub genes via the Cytoscape 3.9.0 cytoHubba plugin, identified 6 common hub genes, namely, AKT1, SRC, STAT3, EGFR, HSP90AA1, and MDM2 via a Venn diagram (Fig. 8B), and constructed a PPI network via Cytoscape 3.9.0 (Fig. 8C). We also performed GO and KEGG enrichment analyses on 44 common targets. GO enrichment revealed that the inhibition of EV71 replication by AST-IV was associated with the response to drugs, thecellular response to external stimuli, the regulation of lipid metabolic process, regulation of reactive oxygen species biosynthetic process, cellular response to oxidative stress, regulation of purine nucleotide metabolic process, activation of protein kinase B activity, regulation of purine nucleotide biosynthetic process, apical plasma membrane (Fig. 8D). KEGG enrichment analysis indicated that the hub genes were mainly enriched in PI3K- AKT signaling pathway, cAMP signaling pathway, and lipid and atherosclerosis signaling pathway (Fig. 8E). To further identify more important target proteins and pathways, we performed KEGG analysis using Metascape (https://Metascape.org/), and key targets were closely related to the PI3K-AKT pathway (Fig. 8F).

### The effects of AST-IV on the PI3K-AKT pathway in EV71-infected and AST-IV-treated GES-1 cells

**Fig. 9:**
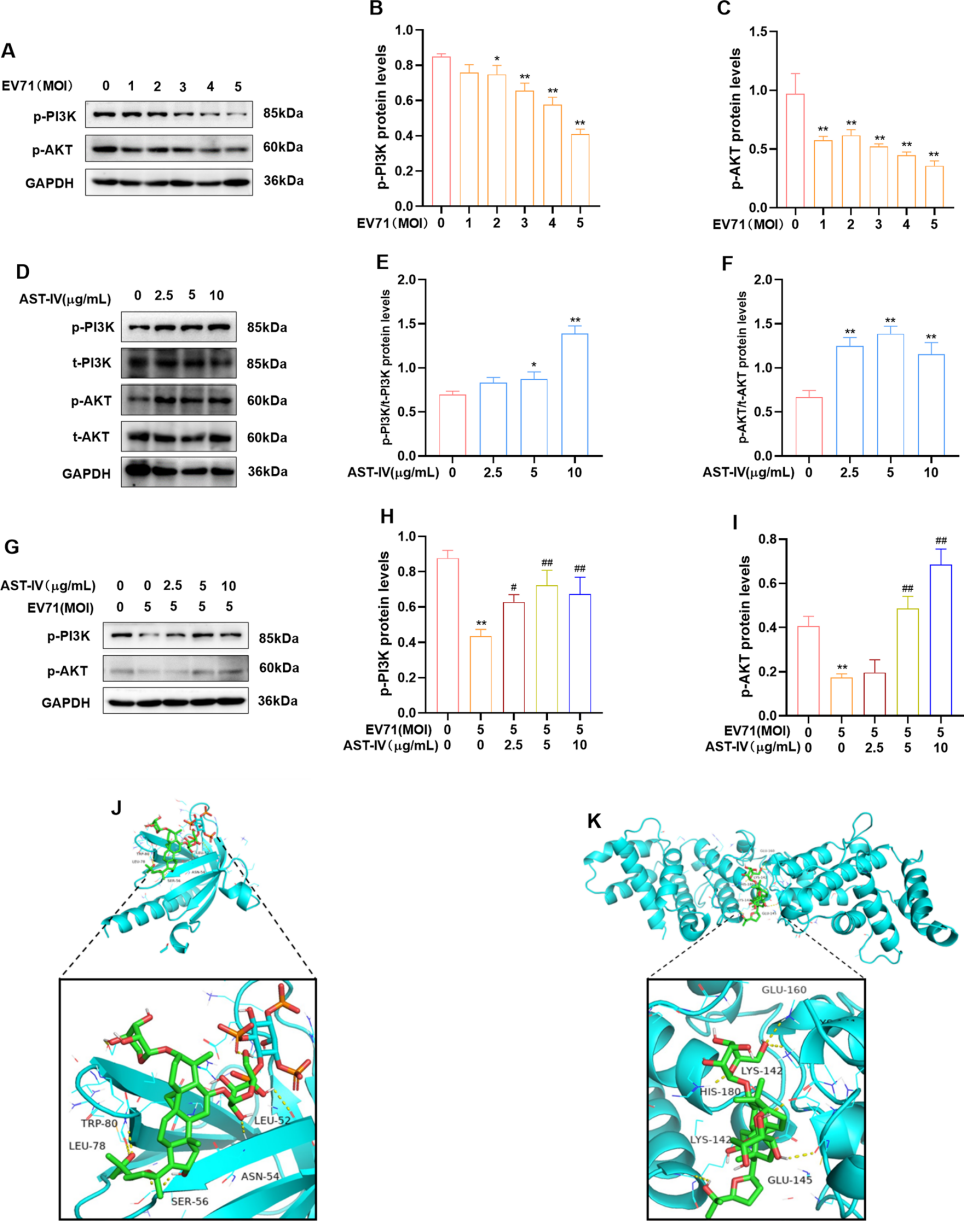
The effects of AST-IV on the PI3K-AKT pathway in EV71-infected and AST-IV-treated GES-1 cells. (**A-C**) Western blot analysis of p-PI3K and p-AKT levels in EV71-infected GES-1 cells. (**D-F**) The protein levels of p-PI3K, t-PI3K, p-AKT, and t-AKT in AST-IV-treated GES-1 cells were analyzed by western blot. (**G-I**) The protein levels of p-PI3K and p-AKT in EV71-infected and AST-IV-treated GES-1 cells were analyzed by Western blot. (**J, K**) The conformation of the molecular docking between AST-IV and AKT1 or PIK3R1. The data are presented as the mean ± SD (*n* = 3) of at least three independent experiments. *P < 0.05, **P < 0.01, ***P < 0.001 compared with the control group; #*P* < 0.05, ##*P* < 0.01 compared with the EV71-infected group

The PI3K-AKT pathway is involved in the regulation of cellular processes such as apoptosis [33] and viral replication [13]. The effects of AST-IV on the PI3K-AKT pathway were investigated in EV71-infected and AST-IV-treated GES-1 cells. First, GES-1 cells were infected with EV71 at different MOIs (1, 3, and 5) for 24 h. The protein levels of p-PI3K and p-AKT were assessed by Western blot. We observed that the levels of p-PI3K and p-AKT were reduced in a dose-dependent manner in the EV71-infected group compared to those in the control group (Fig. 9A-C). This finding suggested that EV71 infection inhibits the activation of the PI3K-AKT pathway in GES-1 cells. Next, GES-1 cells were treated with AST-IV for 24 h, and the protein levels of p-PI3K, t-PI3K, p-AKT, and t-AKT were detected by Western blot. The results showed that p-PI3K and p-AKT levels were increased in AST-IV-treated GES-1 cells, suggesting that AST-IV activated the PI3K-AKT pathway (Fig. 9D-F). Furthermore, we observed that AST-IV treatment reversed the inhibition of the PI3K-AKT signaling pathway caused by EV71 infection (Fig. 9G-I). This suggests that AST-IV has the ability to counteract the negative effects of EV71 on the PI3K-AKT pathway in GES-1 cells. To further validate the Western blot results, we used a molecular docking method to predict the interactions between AST-IV and AKT1 or PIK3R1. The docked binding energies between AST-IV and the AKT1 and PIK3R1 proteins were − 7.12 ± 0.35 and − 7.47 ± 0.25 kcal/mol, respectively (Fig. 9J, K, S3). This shows that there is good bonding between them (binding energy ≤-5 kcal/mol). In conclusion, our findings indicate that AST-IV treatment activates the PI3K-AKT pathway and reverses its inhibition by EV71 infection in GES-1 cells.

### Associations between metabolites and apparent indicators

**Fig. 10:**
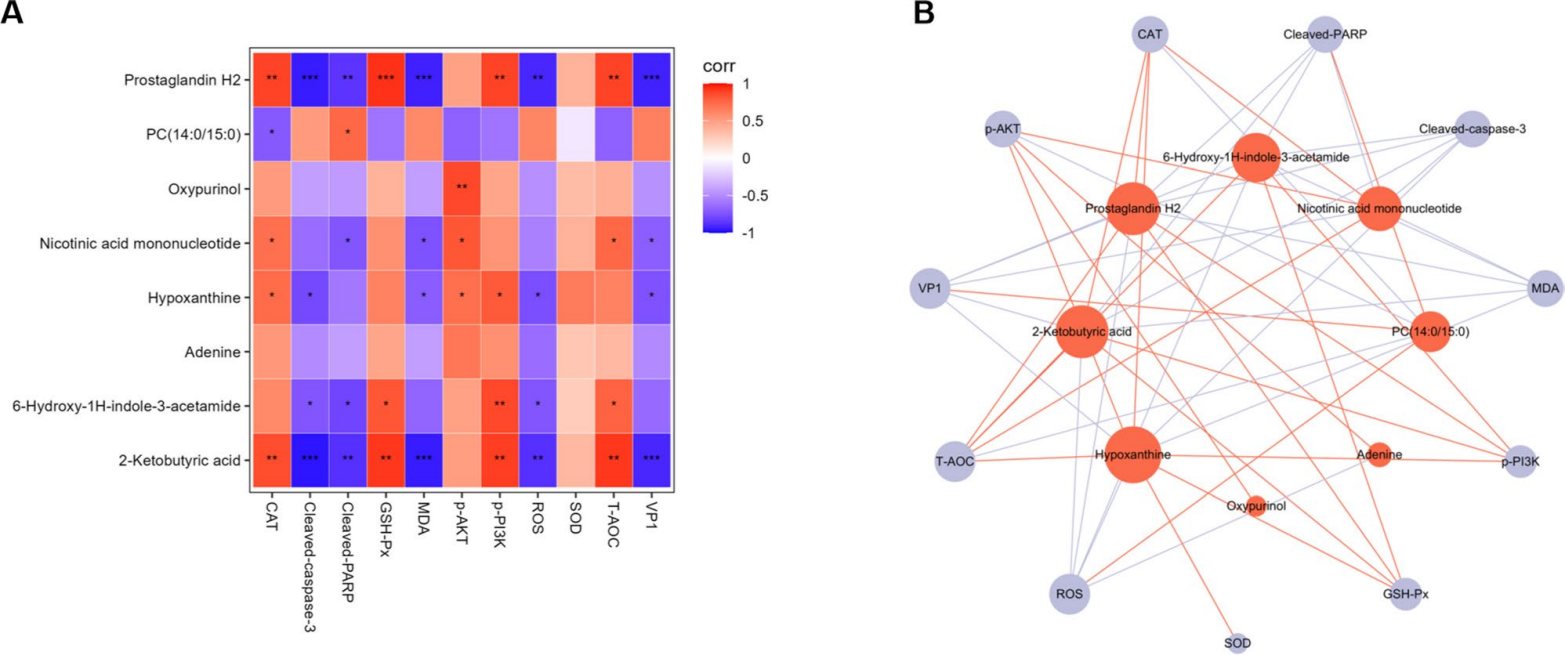
Correlation between targeted metabolites and apparent indicators. (**A**) Association analysis of metabolites with apparent indicators by Spearman’s method. Differences were considered statistically significant at *P* < 0.05. (**B**) Network (only correlations with |R| > 0.6 with *P* < 0.05 were presented) among metabolites and apparent indicators. Red nodes represent differential metabolites, and purple nodes represent apparent indicators. Red lines indicate positive correlations, while purple lines indicate negative correlations

Correlation analyses were performed using Spearman’s algorithm to explore the effects of metabolites on oxidative stress, PI3K-AKT signaling, apoptosis and EV71 virus replication (Fig. 10A). It was shown that hypoxanthine, 2-ketobutyric acid, nicotinic acid mononucleotide, prostaglandin H2, and 6-hydroxy-1 H-indole-3- acetamide were significantly negatively correlated with ROS and MDA. Hypoxanthine, 2-ketobutyric acid, nicotinic acid mononucleotide, prostaglandin H2, and 6-hydroxy-1 H-indole-3- acetamide were significantly positively correlated with T-AOC, GSH-Px, and CAT. Hypoxanthine, 2-ketobutyric acid, nicotinic acid mononucleotide, prostaglandin H2, 6-hydroxy-1 H-indole-3- acetamide, and oxypurinol were significantly positively correlated with PI3K-AKT signaling. Hypoxanthine, 2-ketobutyric acid, nicotinic acid mononucleotide, prostaglandin H2, and 6-hydroxy-1 H-indole-3- acetamide were significantly negatively correlated, and PC (14:0/15:0) was significantly positively correlated with EV71-induced apoptosis. The levels of hypoxanthine, 2-ketobutyric acid, nicotinic acid mononucleotide, and prostaglandin H2 were significantly negatively correlated with the VP1 protein level. To visually represent these correlations, an association network was constructed involving on oxidative stress, PI3K-AKT signaling, apoptosis, EV71 virus replication and differential metabolites (Fig. 10B). In conclusion, these findings indicate that AST-IV may trigger the antioxidant stress response by interacting with eight crucial metabolites to promote PI3K-AKT signaling, thereby suppressing EV71 replication.

## Discussion

We observed that medium and high concentrations of AST-IV intervention in EV71-infected GES-1 cells led to a reduction in VP1 protein levels and viral titers in the supernatant. This finding suggested that AST-IV inhibits EV71 replication. We investigated the mechanism by which AST-IV inhibits EV71 replication via network pharmacology and metabolomics analyses. Network pharmacology indicated that AT-IV may inhibit EV71 replication by AKT1, HSP90 and EGFR et al. core targets to regulate lipid metabolic processes, the cellular response to oxidative stress, purine nucleotide metabolic processes, the PI3K- AKT signaling pathway, the cAMP signaling pathway, and lipid and atherosclerosis signaling pathways. The AKT protein has been shown to be activated in the early stages of EV71 infection [34], and inhibiting the expression of the HSP90 [35] and EGFR [36] proteins can inhibit EV71 replication. Metabolite profiling of the control, EV71, AST-IV, and EV71 and AST-IV groups revealed significant changes in eight key metabolites, including hypoxanthine, 2-ketobutyric acid, adenine, nicotinic acid mononucleotide, prostaglandin H2, oxypurinol, 6-hydroxy-1 H-indole-3-acetamide and PC (14:0/15:0). Importantly, the levels of all eight metabolites were restored to normal cellular levels when AST-IV was applied to EV71-infected cells.

Oxypurinol acts as an inhibitor of xanthine oxidase (XO) and inhibits ROS production and inflammatory responses in bowel diseases [37, 38]. Nicotinic acid mononucleotide (NAMN) exerts anti-hepatitis C virus effects and inhibits ROS production by synthesizing NAD and increasing the NAD/NADH ratio [39, 40]. 6-Hydroxy-1 H-indole-3-acetamide is a precursor for growth hormone synthesis. The growth hormone analog 2,4-dichlorophenoxyacetic acid has been shown to inhibit ROS production and influenza virus replication [41]. These three metabolites were all upregulated in AST-IV-treated GES-1 cells, and inhibited ROS production.

ROS, whether exogenously or endogenously overproduced, can lead to an imbalance in redox homeostasis [42]. Although elevated levels of virus-induced ROS trigger innate antiviral immunity, a recent study reported that ROS production is beneficial for viral replication [43]. This phenomenonhas been observed for alphaviruses and flaviviruses, which exhibit enhanced replication in an oxidized cellular environment [44]. In addition, elevated levels of ROS have been detected in SARS-CoV-2-infected patients, and early diagnosis of COVID-19 can be achieved through real-time detection of ROS levels in fresh sputum by electrochemical tracing [45]. Nrf2, an antioxidant gene, inhibits the replication of vesicular stomatitis virus (VSV) and influenza A virus (H1N1) by suppressing virus-induced oxidative stress [8, 46]. In the context of EV71 infection, the virus was found to induce mitochondrial oxidative stress to promote its own replication [47]. In our study, we observed that EV71 infection caused an increase in cellular ROS production. However, intervention with AST-IV reduced the ROS levels caused by EV71 infection, and the ROS inhibitor NAC inhibited EV71 replication. Moreover, AST-IV treatment of EV71-infected GES-1 cells or RD cells restored the levels of oxidative stress-related markers such as CAT, GSH-Px, T-AOC, and MAD to normal levels. These findings suggest that AST-IV may inhibit EV71 replication by upregulating the levels of oxypurinol, NAMN, and 6-Hydroxy-1 H-indole-3-acetamide to activate the body’s antioxidant system. In summary, AST-IV appears to exert antiviral effects against EV71 infection by reducing ROS production and restoring the antioxidant system.

Hypoxanthine, a nucleotide produced from purines, is an important intermediate in the anabolism of purine nucleotides. The novel C-2 modified hypoxanthine has excellent anti-RNA viral activity [48]. PC (14:0/15:0) is an important component of the bilayer membrane structure of the cell membrane. Viruses can use the cell membrane as a vehicle for noncleavage transport out of the cell and spread to other cells or susceptible hosts to facilitate the spread of infection [49]. The levels of 2-ketobutyric acid are elevated after HIV infection and return to normal after treatment, suggesting that 2-ketobutyric acid plays an important role in the pathogenesis of HIV [50]. In our study, we found that EV71 infection downregulated 2-ketobutyric acid and hypoxanthine levels and upregulated PC (14:0/15:0) levels. All of these metabolites returned to normal cellular levels after AST-IV intervention, suggesting that AST-IV may inhibit EV71 replication and spread by modulating these metabolites.

Prostaglandin E (2) (PEG2) is converted from prostaglandin H2, which can inhibit HIV replication by activating PKA and cAMP [51]. Adenine also activates cAMP [52]. Apoptosis is a common response to viral infection [53–55]. Research suggests that viruses tend to exploit host cells to induce apoptosis in tissues or immune cells, allowing the virus to persist during infection [56]. The EV71 [57] and IAV [58] viruses induce apoptosis in infected cells, allowing the release of daughter viruses to infect neighboring cells. In this study, AST-IV was found to inhibit EV71-induced apoptosis. The PI3K-AKT pathway is a key signaling pathway involved in the regulation of viral infection [12] and apoptosis [59]. Research has shown that inhibition of the PI3K-AKT pathway can promote mammalian reovirus replication [60], while upregulation of the PI3K-AKT pathway can suppress endoplasmic reticulum stress-induced cell apoptosis [61]. Both cAMP and ROS can regulate the PI3K-AKT pathway, with cAMP activating it [62] and ROS inhibiting it [63]. Therefore, we focused on the PI3K-AKT pathway as the key pathway through which AST-IV exerts its anti-EV71 effect. The PI3K-AKT pathway is activated during the early stages of EV71 infection but inhibited after 1 h of infection [64]. In our study, the PI3K-AKT pathway was inhibited in EV71-infected GES-1 cells for 24 h, whereas AST-IV treatment restored its activity. This study indicates that AST-IV activates the cAMP pathway and the body’s antioxidant system by modulating these metabolites, which further activates the PI3K-AKT pathway, thereby inhibiting EV71-induced cell.

apoptosis and replication of EV71. In our future studies, we will first experimentally determine the targeted binding of AST-IV to PI3K and AKT. Second, we aim to validate these findings in animal models and potentially in clinical trials to assess the translational potential of AST-IV in the treatment of EV71 infection.

## Conclusion

In conclusion, our research revealed that AST-IV can effectively inhibit EV71 replication. We propose that AST-IV may exert its anti-EV71 effects by targeting eight metabolites. These metabolites are likely to activate the body’s antioxidant system, which further activates the PI3K-AKT pathway to inhibit EV71-induced cell apoptosis and EV71 replication.

### Electronic supplementary material

Below is the link to the electronic supplementary material.

Supplementary Material 1

Supplementary Material 2

## Data Availability

The datasets supporting the conclusions of this article are included within the article. Further inquiries can be addressed to the corresponding author.

## References

[1] Cui G, Wang H, Yang C, Zhou X, Wang J, Wang T, Ma T (2022). Berberine prevents lethal EV71 neurological infection in newborn mice. Front Pharmacol.

[2] Ohka S, Tan SH, Ishiyama E, Ogasawara K, Hanasaka T, Ishida K, Hagiwara K, Liu CC, Chong PC, Hanaki KI, Schiavo G. The uncoating of EV71 in mature late endosomes requires CD-M6PR. Biol Open 2022, 11. 10.1242/bio.059469 PMC9493940 35929543

[3] Nguyen TT, Chiu CH, Lin CY, Chiu NC, Chen PY, Le TTV, Le DN, Duong AH, Nguyen VL, Huynh TN (2022). Efficacy, safety, and immunogenicity of an inactivated, adjuvanted enterovirus 71 vaccine in infants and children: a multiregion, double-blind, randomised, placebo-controlled, phase 3 trial. Lancet.

[4] Zhan X, Wu R, Kong XH, You Y, He K, Sun XY, Huang Y, Chen WX, Duan L (2023). Elevated neutrophil extracellular traps by HBV-mediated S100A9-TLR4/RAGE-ROS cascade facilitate the growth and metastasis of hepatocellular carcinoma. Cancer Commun (Lond).

[5] Jadaun P, Shah P, Harshithkumar R, Said MS, Bhoite SP, Bokuri S, Ravindran S, Mishra N, Mukherjee A (2023). Antiviral and ROS scavenging potential of Carica papaya Linn and Psidium guajava leaves extract against HIV-1 infection. BMC Complement Med Ther.

[6] Sun Q, Ye Z, Qin Y, Fan G, Ji S, Zhuo Q, Xu W, Liu W, Hu Q, Liu M (2020). Oncogenic function of TRIM2 in pancreatic cancer by activating ROS-related NRF2/ITGB7/FAK axis. Oncogene.

[7] Olagnier D, Farahani E, Thyrsted J, Blay-Cadanet J, Herengt A, Idorn M, Hait A, Hernaez B, Knudsen A, Iversen MB (2020). SARS-CoV2-mediated suppression of NRF2-signaling reveals potent antiviral and anti-inflammatory activity of 4-octyl-itaconate and dimethyl fumarate. Nat Commun.

[8] Wang H, Jia X, Zhang M, Cheng C, Liang X, Wang X, Xie F, Wang J, Yu Y, He Y (2023). Isoliquiritigenin inhibits virus replication and virus-mediated inflammation via NRF2 signaling. Phytomedicine.

[9] Long F, Zhang M, Yang X, Liang X, Su L, An T, Zhang G, Zeng Z, Liu Y, Chen W, Chen J (2022). The Antimalaria Drug Artesunate inhibits Porcine Reproductive and Respiratory Syndrome Virus replication by activating AMPK and Nrf2/HO-1 signaling pathways. J Virol.

[10] Dunn EF, Connor JH (2012). HijAkt: the PI3K/Akt pathway in virus replication and pathogenesis. Prog Mol Biol Transl Sci.

[11] Li F, Li J, Wang PH, Yang N, Huang J, Ou J, Xu T, Zhao X, Liu T, Huang X (2021). SARS-CoV-2 spike promotes inflammation and apoptosis through autophagy by ROS-suppressed PI3K/AKT/mTOR signaling. Biochim Biophys Acta Mol Basis Dis.

[12] Lahon A, Arya RP, Banerjea AC (2021). Dengue Virus Dysregulates Master transcription factors and PI3K/AKT/mTOR signaling pathway in Megakaryocytes. Front Cell Infect Microbiol.

[13] Zhang X, Ming Y, Fu X, Niu Y, Lin Q, Liang H, Luo X, Liu L, Li N (2022). PI3K/AKT/p53 pathway inhibits infectious spleen and kidney necrosis virus infection by regulating autophagy and immune responses. Fish Shellfish Immunol.

[14] Wang Z, Wu Y, Pei C, Wang M, Wang X, Shi S, Huang D, Wang Y, Li S, Xiao W (2022). Astragaloside IV pre-treatment attenuates PM2.5-induced lung injury in rats: impact on autophagy, apoptosis and inflammation. Phytomedicine.

[15] Cheng BH, Zhou X, Wang Y, Chan JY, Lin HQ, Or PM, Wan DC, Leung PC, Fung KP, Wang YF, Lau CB (2015). Herb-drug interaction between an anti-HIV Chinese herbal SH formula and atazanavir in vitro and in vivo. J Ethnopharmacol.

[16] Yeh YC, Doan LH, Huang ZY, Chu LW, Shi TH, Lee YR, Wu CT, Lin CH, Chiang ST, Liu HK (2021). Honeysuckle (Lonicera japonica) and Huangqi (Astragalus membranaceus) suppress SARS-CoV-2 entry and COVID-19 related cytokine storm in Vitro. Front Pharmacol.

[17] Tang JL, Xin M, Zhang LC (2022). Protective effect of Astragalus membranaceus and astragaloside IV in sepsis-induced acute kidney injury. Aging.

[18] Liu Z, Zhou Z, Ai P, Zhang C, Chen J, Wang Y (2022). Astragaloside IV attenuates ferroptosis after subarachnoid hemorrhage via Nrf2/HO-1 signaling pathway. Front Pharmacol.

[19] Zhang Y, Zhu H, Huang C, Cui X, Gao Y, Huang Y, Gong W, Zhao Y, Guo S (2006). Astragaloside IV exerts antiviral effects against coxsackievirus B3 by upregulating interferon-gamma. J Cardiovasc Pharmacol.

[20] Indu P, Arunagirinathan N, Rameshkumar MR, Sangeetha K, Divyadarshini A, Rajarajan S (2021). Antiviral activity of astragaloside II, astragaloside III and astragaloside IV compounds against dengue virus: computational docking and in vitro studies. Microb Pathog.

[21] Wang S, Li J, Huang H, Gao W, Zhuang C, Li B, Zhou P, Kong D (2009). Anti-hepatitis B virus activities of astragaloside IV isolated from radix astragali. Biol Pharm Bull.

[22] Shang L, Qu Z, Sun L, Wang Y, Liu F, Wang S, Gao H, Jiang F (2011). Astragaloside IV inhibits adenovirus replication and apoptosis in A549 cells in vitro. J Pharm Pharmacol.

[23] Liu T, Yang F, Liu J, Zhang M, Sun J, Xiao Y, Xiao Z, Niu H, Ma R, Wang Y (2019). Astragaloside IV reduces cardiomyocyte apoptosis in a murine model of coxsackievirus B3-induced viral myocarditis. Exp Anim.

[24] Han Y, Guo S, Li Y, Li J, Zhu L, Liu Y, Lv Y, Yu D, Zheng L, Huang C (2023). Berberine ameliorate inflammation and apoptosis via modulating PI3K/AKT/NFκB and MAPK pathway on dry eye. Phytomedicine.

[25] Park JS, Mathison BD, Hayek MG, Zhang J, Reinhart GA, Chew BP (2013). Astaxanthin modulates age-associated mitochondrial dysfunction in healthy dogs. J Anim Sci.

[26] Huang ML, Chiang PS, Luo ST, Liou GY, Lee MS (2010). Development of a high-throughput assay for measuring serum neutralizing antibody against enterovirus 71. J Virol Methods.

[27] Zhang X, Hao J, Sun C, Du J, Han Q, Li Q (2022). Total astragalosides decrease apoptosis and pyroptosis by inhibiting enterovirus 71 replication in gastric epithelial cells. Exp Ther Med.

[28] Chin CH, Chen SH, Wu HH, Ho CW, Ko MT, Lin CY (2014). cytoHubba: identifying hub objects and sub-networks from complex interactome. BMC Syst Biol.

[29] Pellegrini FR, De Martino S, Fianco G, Ventura I, Valente D, Fiore M, Trisciuoglio D, Degrassi F (2023). Blockage of autophagosome-lysosome fusion through SNAP29 O-GlcNAcylation promotes apoptosis via ROS production. Autophagy.

[30] Li J, Li Y, Wang X, Xie Y, Lou J, Yang Y, Jiang S, Ye M, Chen H, Diao W, Xu S (2023). Pinocembrin alleviates pyroptosis and apoptosis through ROS elimination in random skin flaps via activation of SIRT3. Phytother Res.

[31] Xu XQ, Xu T, Ji W, Wang C, Ren Y, Xiong X, Zhou X, Lin SH, Xu Y, Qiu Y (2023). Herpes simplex virus 1-Induced Ferroptosis contributes to viral encephalitis. mBio.

[32] Deng L, Liang Y, Ariffianto A, Matsui C, Abe T, Muramatsu M, Wakita T, Maki M, Shibata H, Shoji I, Hepatitis C, Virus-Induced (2022). ROS/JNK signaling pathway activates the E3 ubiquitin ligase itch to promote the release of HCV particles via polyubiquitylation of VPS4A. J Virol.

[33] Fang Z, Yushanjiang F, Wang G, Zheng X, Jiang X (2023). Germacrone mitigates cardiac remodeling by regulating PI3K/AKT-mediated oxidative stress, inflammation, and apoptosis. Int Immunopharmacol.

[34] Wong WR, Chen YY, Yang SM, Chen YL, Horng JT (2005). Phosphorylation of PI3K/Akt and MAPK/ERK in an early entry step of enterovirus 71. Life Sci.

[35] Zhu G, Wu C, Wang Q, Deng D, Lin B, Hu X, Qiu F, Li Z, Huang C, Yang Q, Zhang D (2023). Antiviral activity of the HSP90 inhibitor VER-50589 against enterovirus 71. Antiviral Res.

[36] Zhang L, Chen X, Shi Y, Zhou B, Du C, Liu Y, Han S, Yin J, Peng B, He X, Liu W (2014). miR-27a suppresses EV71 replication by directly targeting EGFR. Virus Genes.

[37] Fang J, Yin H, Liao L, Qin H, Ueda F, Uemura K, Eguchi K, Bharate GY, Maeda H (2016). Water soluble PEG-conjugate of xanthine oxidase inhibitor, PEG-AHPP micelles, as a novel therapeutic for ROS related inflammatory bowel diseases. J Control Release.

[38] López-Iranzo FJ, López-Rodas AM, Franco L, López-Rodas G (2020). Pentoxifylline and Oxypurinol: potential drugs to prevent the Cytokine Release (storm) syndrome caused by SARS-CoV-2?. Curr Pharm Des.

[39] Wang Z, Gao Y, Zhang C, Hu H, Guo D, Xu Y, Xu Q, Zhang W, Deng S, Lv P (2017). Quinolinate Phosphoribosyltransferase is an Antiviral Host Factor against Hepatitis C Virus Infection. Sci Rep.

[40] Zwilling M, Theiss C, Matschke V. Caffeine and NAD(+) improve motor neural Integrity of dissociated wobbler cells in Vitro. Antioxid (Basel) 2020, 9. 10.3390/antiox9060460 PMC7346375 32471290

[41] Enkhtaivan G, Muthuraman P, Kim DH. Inhibitory effect of 2,4-dichlorophenoxyacetic acid on ROS, autophagy formation, and mRNA replication for influenza virus infection. J Mol Recognit 2017, 30. 10.1002/jmr.2616 28233349

[42] Xiang K, Wu H, Liu Y, Wang S, Li X, Yang B, Zhang Y, Ma L, Lu G, He L (2023). MOF-derived bimetallic nanozyme to catalyze ROS scavenging for protection of myocardial injury. Theranostics.

[43] Foo J, Bellot G, Pervaiz S, Alonso S (2022). Mitochondria-mediated oxidative stress during viral infection. Trends Microbiol.

[44] Gullberg RC, Jordan Steel J, Moon SL, Soltani E, Geiss BJ (2015). Oxidative stress influences positive strand RNA virus genome synthesis and capping. Virology.

[45] Miripour ZS, Sarrami-Forooshani R, Sanati H, Makarem J, Taheri MS, Shojaeian F, Eskafi AH, Abbasvandi F, Namdar N, Ghafari H (2020). Real-time diagnosis of reactive oxygen species (ROS) in fresh sputum by electrochemical tracing; correlation between COVID-19 and viral-induced ROS in lung/respiratory epithelium during this pandemic. Biosens Bioelectron.

[46] Waqas FH, Shehata M, Elgaher WAM, Lacour A, Kurmasheva N, Begnini F, Kiib AE, Dahlmann J, Chen C, Pavlou A (2023). NRF2 activators inhibit influenza a virus replication by interfering with nucleo-cytoplasmic export of viral RNPs in an NRF2-independent manner. PLoS Pathog.

[47] Cheng ML, Weng SF, Kuo CH, Ho HY (2014). Enterovirus 71 induces mitochondrial reactive oxygen species generation that is required for efficient replication. PLoS ONE.

[48] Nair V, Ussery MA (1992). New hypoxanthine nucleosides with RNA antiviral activity. Antiviral Res.

[49] Altan-Bonnet N (2017). Lipid tales of viral replication and transmission. Trends Cell Biol.

[50] Kaur SU, Oyeyemi BF, Shet A, Gopalan BP, Bhavesh DH, Tandon NS (2020). Plasma metabolomic study in perinatally HIV-infected children using 1H NMR spectroscopy reveals perturbed metabolites that sustain during therapy. PLoS ONE.

[51] Hayes MM, Lane BR, King SR, Markovitz DM, Coffey MJ (2002). Prostaglandin E(2) inhibits replication of HIV-1 in macrophages through activation of protein kinase A. Cell Immunol.

[52] Zhang Y, Wang X, Wu Q, Wang H, Zhao L, Wang X, Mu M, Xie E, He X, Shao D (2018). Adenine alleviates iron overload by cAMP/PKA mediated hepatic hepcidin in mice. J Cell Physiol.

[53] Yang Y, Wu Y, Meng X, Wang Z, Younis M, Liu Y, Wang P, Huang X (2022). SARS-CoV-2 membrane protein causes the mitochondrial apoptosis and pulmonary edema via targeting BOK. Cell Death Differ.

[54] Mundhra S, Bondre VP (2023). Higher replication potential of West Nile virus governs apoptosis induction in human neuroblastoma cells. Apoptosis.

[55] Li JJ, Liu ML, Lv JN, Chen RL, Ding K, He JQ (2022). Polysaccharides from Platycodonis Radix ameliorated respiratory syncytial virus-induced epithelial cell apoptosis and inflammation through activation of miR-181a-mediated Hippo and SIRT1 pathways. Int Immunopharmacol.

[56] Iannello A, Debbeche O, Martin E, Attalah LH, Samarani S, Ahmad A (2006). Viral strategies for evading antiviral cellular immune responses of the host. J Leukoc Biol.

[57] Cong H, Du N, Yang Y, Song L, Zhang W, Tien P (2016). Enterovirus 71 2B induces cell apoptosis by directly inducing the conformational activation of the proapoptotic protein Bax. J Virol.

[58] Ampomah PB, Lim LHK (2020). Influenza a virus-induced apoptosis and virus propagation. Apoptosis.

[59] Peng X, Guo H, Zhang X, Yang Z, Ruganzu JB, Yang Z, Wu X, Bi W, Ji S, Yang W (2023). TREM2 inhibits tau hyperphosphorylation and neuronal apoptosis via the PI3K/Akt/GSK-3β signaling pathway in vivo and in vitro. Mol Neurobiol.

[60] Tian J, Zhang X, Wu H, Liu C, Li Z, Hu X, Su S, Wang LF, Qu L (2015). Blocking the PI3K/AKT pathway enhances mammalian reovirus replication by repressing IFN-stimulated genes. Front Microbiol.

[61] Wang S, Duan J, Liao J, Wang Y, Xiao X, Li L, Liu Y, Gu H, Yang P, Fu D (2022). LncRNA H19 inhibits ER stress induced apoptosis and improves diabetic cardiomyopathy by regulating PI3K/AKT/mTOR axis. Aging.

[62] Farid HA, Sayed RH, El-Shamarka ME, Abdel-Salam OME, El Sayed NS. PI3K/AKT signaling activation by roflumilast ameliorates rotenone-induced Parkinson’s disease in rats. Inflammopharmacology 2023. 10.1007/s10787-023-01305-x PMC11006765 37541971

[63] Zhang L, Zhang X, Che D, Zeng L, Zhang Y, Nan K, Zhang X, Zhang H, Guo Z (2023). 6-Methoxydihydrosanguinarine induces apoptosis and autophagy in breast cancer MCF-7 cells by accumulating ROS to suppress the PI3K/AKT/mTOR signaling pathway. Phytother Res.

[64] Zhang H, Cong H, Song L, Tien P (2014). The nuclear protein Sam68 is redistributed to the cytoplasm and is involved in PI3K/Akt activation during EV71 infection. Virus Res.

