# Supplementary material for: Metabolomics combined with network pharmacology reveals a role for astragaloside IV in inhibiting enterovirus 71 replication via PI3K-AKT signaling
Source: J Transl Med. 2024 Jun 10;22:555. doi: 10.1186/s12967-024-05355-9 (PMC11163744; doi:10.1186/s12967-024-05355-9)
Supplement: Supplementary file 1 — Supplementary Material 1 [file 12967_2024_5355_MOESM1_ESM.docx]

**Metabolomics combined with network pharmacology reveals a role for astragaloside IV in inhibiting enterovirus 71 replication via PI3K-AKT signaling**


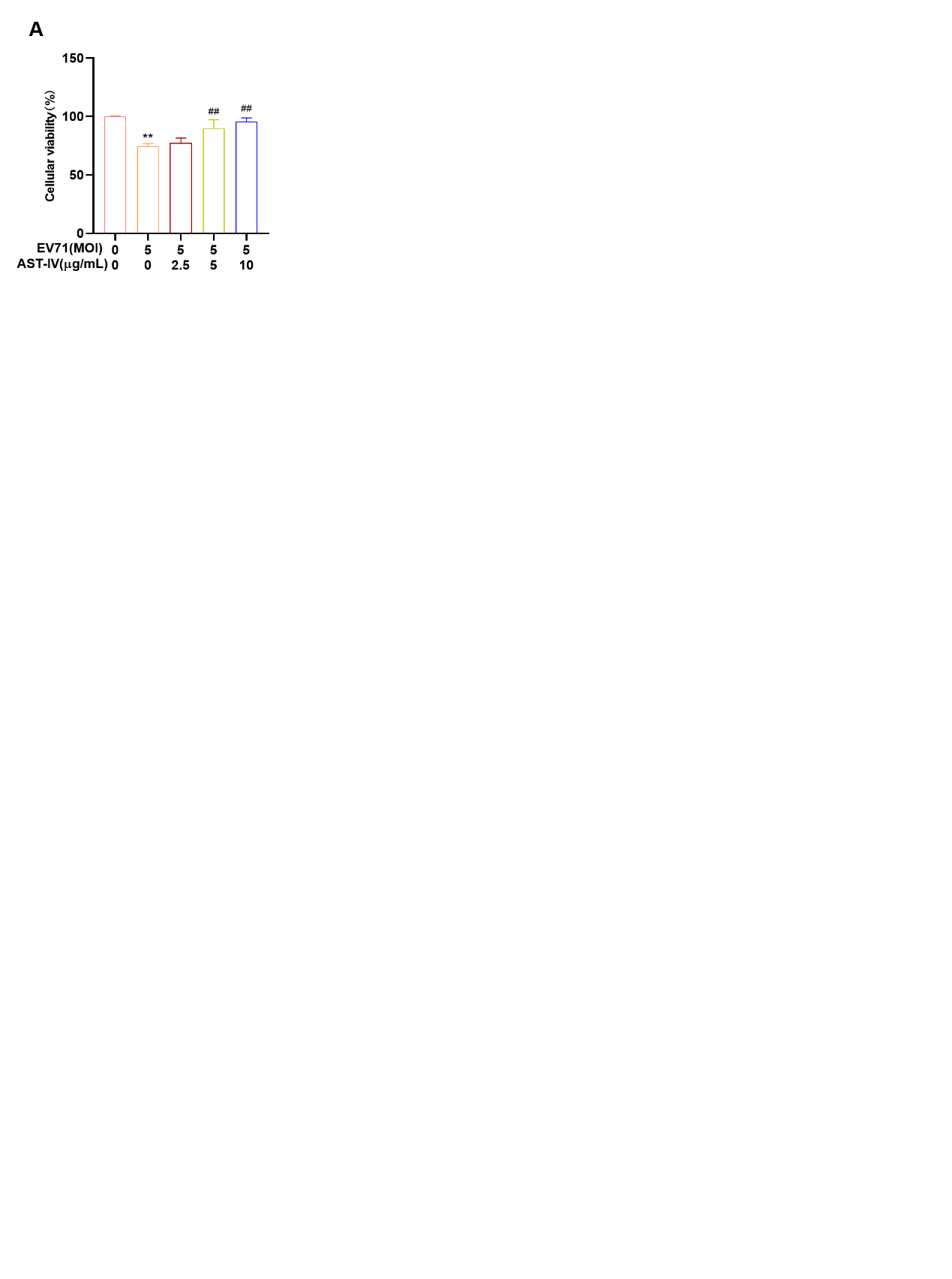


Fig. S1. AST-IV increased the activity of EV71 infected RD cells. (A) The CCK-8 assay was used to measure the viability of RD cells in the different treatment groups.


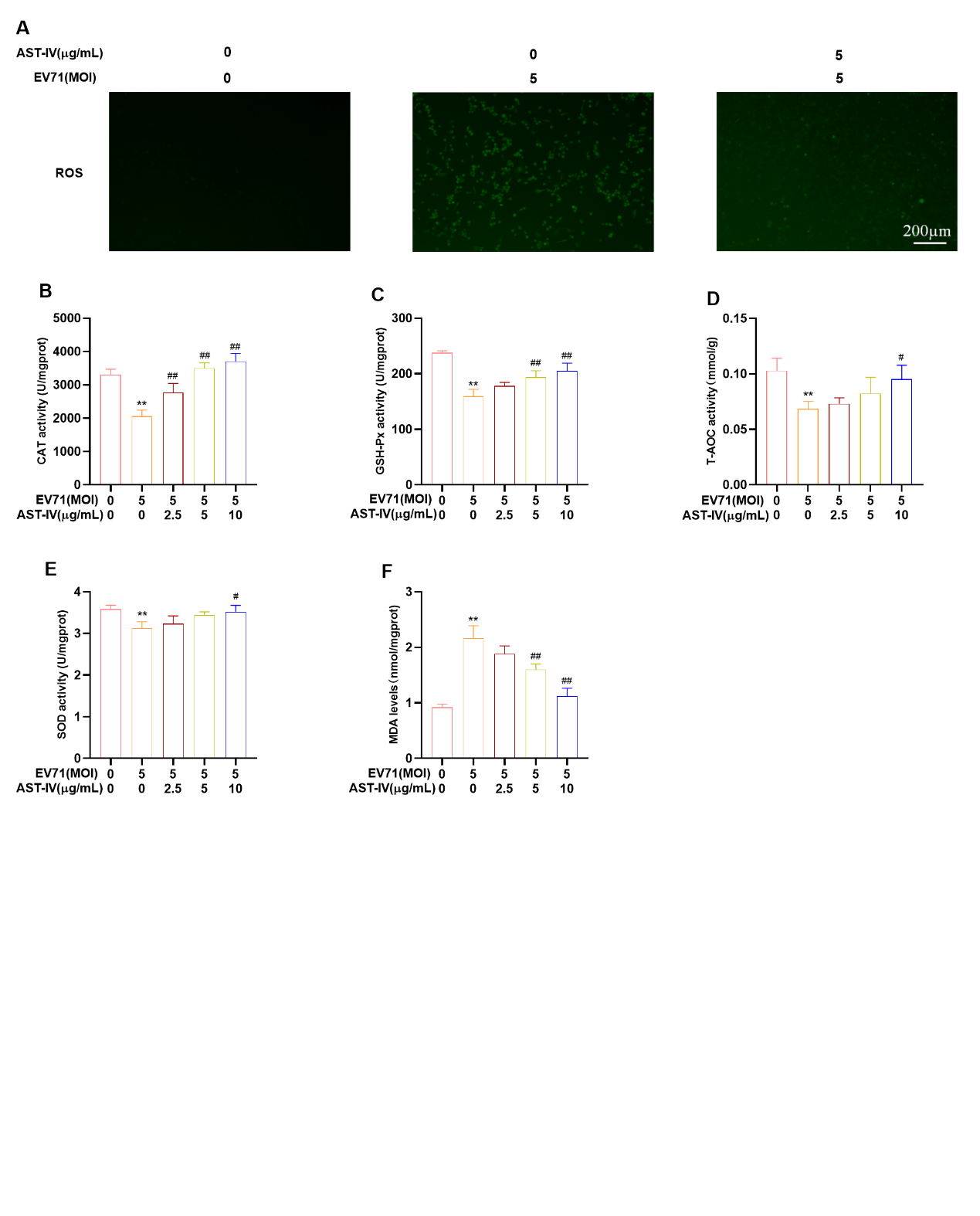


Fig. S2. Effects of AST-IV on antioxidative activity. (A) ROS levels, (B) CAT activity, (C) GSH-Px activity, (D) T-AOC activity, (E) SOD activity, and (F) MDA levels were measured in different treatment groups in RD cells.
