# Supplementary material for: Metabolomics combined with network pharmacology reveals a role for astragaloside IV in inhibiting enterovirus 71 replication via PI3K-AKT signaling
Source: J Transl Med. 2024 Jun 10;22:555. doi: 10.1186/s12967-024-05355-9 (PMC11163744; doi:10.1186/s12967-024-05355-9)
Supplement: Supplementary file 2 — Supplementary Material 2 [file 12967_2024_5355_MOESM2_ESM.docx]

Dear Editor and Professors,

We have carefully read the comments of the reviewers. Thank you very much for helping us to point out the problems, so that the manuscript can be further improved. In view of your valuable comments, we have thought one by one and made the following explanation and revision.

Reviewer #1:

The main concern regards the use of one only cell line to develop all experiments. In Method section the authors report normal human gastric epithelial (GES-1) and human rhabdomyosarcoma (RD) cells. In results section they reported all the data only on GES-1 cells. To validate their results, the authors should repeat at least the initial experiments (cell viability assay, ROS measurement, antioxidant activity) on at least one other cell line. It is understable that metabolomic studies are more complicated and expensive and therefore working on many cell lines is labor-intensive and expensible but in the case of other experiments, as those reported above, this problem does not exist and therefore it is advisable to add other experiments in the paper. Only in this way it is possible to validate the obtained results on GES-1 cells.

**Reply:** Thank you for your professional advice. In our original manuscript, the RD cells were used to virus amplification and performed TCID50 assay, and the GES-1 cells were used to experimental verification. We have repeated the initial experiments (cell viability assay, ROS measurement, antioxidant activity) on the RD cells and the results showed that AST-IV increased the viability of EV71-infected RD cells, reduced ROS levels and increased the activity of CAT, GSH-Px, and T-AOC while decreasing MDA levels in EV71-infected RD cells. These results were consistent with those observed in GES-1 cells. We have included this additional data in the supplementary materials section of the revised manuscript. The revised results are as follows:

Fig. S1.

Fig. S2.

Minor concerns:

- In "LC-MSMS method for GES-1 cell metabolomics" paragraph the authors report the use of extraction solution. What is meant? The authors should add more details.

**Reply:** Thank you for your advice. The extraction solution of LC-MSMS method was a mixture of methanol (MeOH), acetonitrile (ACN), and water in a ratio of 2:2:1 (v/v) and we have included this detail in the manuscript. The revised manuscript is as follows:

- About docking method, the authors should add details about grid dimension, number of step, the amino acids used to define the grid, number of complexes generated by Autodock. Did the authors perform rigid or flexible protocol?

**Reply:** Thank you for your suggestion. We have added additional details about the docking in the method section of the manuscript. The revised manuscript is as follows:

- How do the authors think to continue their studies? How will they use these results in translational studies? The authors should add comments regarding these points.

**Reply:** Thank you for your insightful questions. In our future studies, we will first experimentally determine the targeted binding of AST-IV to PI3K and AKT. Second, we aim to validate these findings in animal models and potentially in clinical trials to assess the translational potential of AST-IV in the treatment of EV71 infection. We have added it in the discussion. The revised manuscript is as follows:

Reviewer #2:

The scientific work produced by J. Hao and colleagues is of particular interest and can be considered acceptable. As a minor revision, it is requested to discuss in the discussion section the pathways identified in the bioinformatics analysis by the authors, well-known as HSP90, EGFR, AKT1, and describe their potential role in the context of the presented work.

**Reply:** Thank you for your valuable suggestion. We have included a discussion on the pathways identified in the bioinformatics analysis, specifically focusing on HSP90, EGFR, and AKT1 as potential targets of AST-IV in the treatment of hand-foot-and-mouth disease. The revised manuscript is as follows:

In addition to the above modifications, we added scales to the PI images of Fig. 2A and ROS images of Fig. 6A. The revised manuscript is as follows:

Fig. 2A

Fig. 6A
